# Amifostine attenuates bleomycin-induced pulmonary fibrosis in mice through inhibition of the PI3K/Akt/mTOR signaling pathway

**DOI:** 10.1038/s41598-023-34060-8

**Published:** 2023-06-28

**Authors:** Wenting Yang, Lin Pan, Yiju Cheng, Xiao Wu, Songsong Huang, Juan Du, Honglan Zhu, Menglin Zhang, Yuquan Zhang

**Affiliations:** 1 Department of Respiratory and Critical Care Medicine, The Affiliated Hospital of Guizhou Medical University, Guiyang, 550004 China; 2 Department of Respiratory and Critical Care Medicine, The First People’s Hospital of Guiyang, Guiyang, 550004 China; 3 Guizhou Medical University, Guiyang, 550004 China; 4 Department of Pathology, The Affiliated Hospital of Guizhou Medical University, Guiyang, 550004 China

**Keywords:** Cancer, Drug discovery, Stem cells, Biomarkers, Diseases, Health care, Health occupations, Medical research, Molecular medicine, Oncology, Pathogenesis, Rheumatology, Risk factors, Signs and symptoms

## Abstract

Amifostine is a normal cell protection agent, not only used in the adjuvant therapy of lung cancer, ovarian cancer, breast cancer, nasopharyngeal cancer, bone tumor, digestive tract tumor, blood system tumor and other cancers in order to reduce the toxicity of chemotherapy drugs, and recent studies have reported that the drug can also reduce lung tissue damage in patients with pulmonary fibrosis, but its mechanism of action is not yet fully understood. In this study, we explored the potential therapeutic effects and molecular mechanisms of AMI on bleomycin (BLM)-induced pulmonary fibrosis in mice. A mouse model of pulmonary fibrosis was established using BLM. We then assessed histopathological changes, inflammatory factors, oxidative indicators, apoptosis, epithelial-mesenchymal transition, extracellular matrix changes, and levels of phosphatidylinositol 3-kinase (PI3K)/Akt/mammalian target of rapamycin (mTOR) signaling pathway-related proteins in the BLM-treated mice to determine the effect of AMI treatment on these factors. BLM-treated mice had substantial lung inflammation and abnormal extracellular matrix deposition. Overall, treatment with AMI significantly improved BLM-induced lung injury and pulmonary fibrosis. More specifically, AMI alleviated BLM-induced oxidative stress, inflammation, alveolar cell apoptosis, epithelial-mesenchymal transition, and extracellular matrix deposition by regulating the PI3K/Akt/mTOR signaling pathway. This finding that AMI can alleviate pulmonary fibrosis in a mouse model by inhibiting activation of the PI3K/Akt/mTOR signaling pathway lays a foundation for potential future clinical application of this agent in patients with pulmonary fibrosis.

## Introduction

Pulmonary fibrosis is a disease with high morbidity and mortality that is characterized by the presence of epithelial cell damage, abnormal proliferation and activation of lung fibroblasts, massive collagen deposition, and abnormal changes in the extracellular matrix (ECM)^1^. Although the US Food and Drug Administration (FDA) has approved the use of pirfenidone and nintedanib for the treatment of pulmonary fibrosis, these agents have not been found to improve the survival rate of patients or to reverse lung function. These agents have also been associated with serious side effects^2^. There is therefore an urgent need for new treatments in this patient population.

As research regarding pulmonary fibrosis has advanced, our understanding of the pathogenesis of this condition has improved. In patients with pulmonary fibrosis, alveolar epithelial injury leads to the activation of various inflammatory responses and signaling pathways, resulting in increased release of profibrotic mediators and thus leading to an imbalance between profibrotic and antifibrotic factors^3–6^. In particular, the differentiation of fibroblasts to myofibroblasts is a critical step in the fibrotic process^7^. Research has shown that myofibroblasts in fibrotic lung tissue exhibit a profibrotic secretory phenotype with abnormal proliferation rates and low rates of apoptosis^8^, leading to increased numbers of ECM components (eg, collagen and fibronectin) and to lung scarring^9^. The EMT is a process in which the differentiated epithelial cells lose their adhesion and epithelial markers and transform into mesenchymal cells, with an increase in the ability of cell migration and invasion. This is an important mechanism leading to pulmonary fibrosis. The epithelial-mesenchymal transition (EMT), which is a source of myofibroblasts, is characterized by increased α-smooth muscle actin (α-SMA) and N-cadherin expression, high proliferative rates, and decreased numbers of epithelial markers such as E-cadherin^10,11^. Phosphatidylinositol 3-kinase (PI3K)/Akt promotes EMT and the expression of α-SMA and is therefore involved in the pathogenesis of fibrosis^12,13^. Interaction between transforming growth factor-β (TGF-β) and PI3K/Akt also promotes the development of pulmonary fibrosis^14^, and activation of PI3K/Akt also promotes activation of downstream targets such as mammalian target of rapamycin (mTOR)^15,16^. The PI3K/Akt/mTOR signaling pathway is therefore an important target for the treatment of pulmonary fibrosis^17^.

Analogues of amifostine: DRDE-30 can protect against radiomimetic and bleomycin-induced lung injury in mice^18^. Studies have shown that the amifostine analogue DRDE-30 exerts its lung protective effect by reducing radiation-induced oxidative stress, cell death, and vascular permeability, as well as inhibiting inflammatory and fibrotic responses in the lungs^19^. Amifostine analogue DRDE-30 ameliorates bleomycin-induced lung injury and pulmonary fibrosis by inhibiting MAPK kinase activation and inhibiting NF-κB-mediated inflammatory response^20^. Amifostine (AMI) is the first clinical radioprotectant to be approved by the FDA. Amifostine is effective in reducing late lung toxicity in mice^21,22^ and is approved as a cytoprotective agent in radiotherapy patients for head and neck cancers^23,24^. Amifortine can alleviate bleomycin-induced pulmonary fibrosis in rats ^25^. AMI has been found to reduce the incidence and severity of radiation pneumonitis^26–28^. Multiple animal and preclinical studies have shown that AMI reduces damage to lung tissue^29^. Previous studies have also shown that down-regulation of TGF-β/SMAD-2 by AMI can inhibit EMT, reduce inflammation, inhibit collagen deposition, and delay pulmonary fibrosis^30^. However, the exact protective mechanism of AMI in pulmonary fibrosis is not fully understood. For instance, it remains unknown whether AMI inhibits pulmonary fibrosis by inhibiting the PI3K/Akt/mTOR pathway. In this study, we sought to investigate the therapeutic effect of AMI on bleomycin (BLM)-induced pulmonary fibrosis in mice. More specifically, we sought to elucidate the possible mechanism of action of AMI by evaluating its effect on oxidative stress, inflammation, apoptosis, EMT, ECM, and related signaling pathways.

## Results

### AMI attenuates BLM-induced lung injury and fibrosis in mice

**Figure 1:**
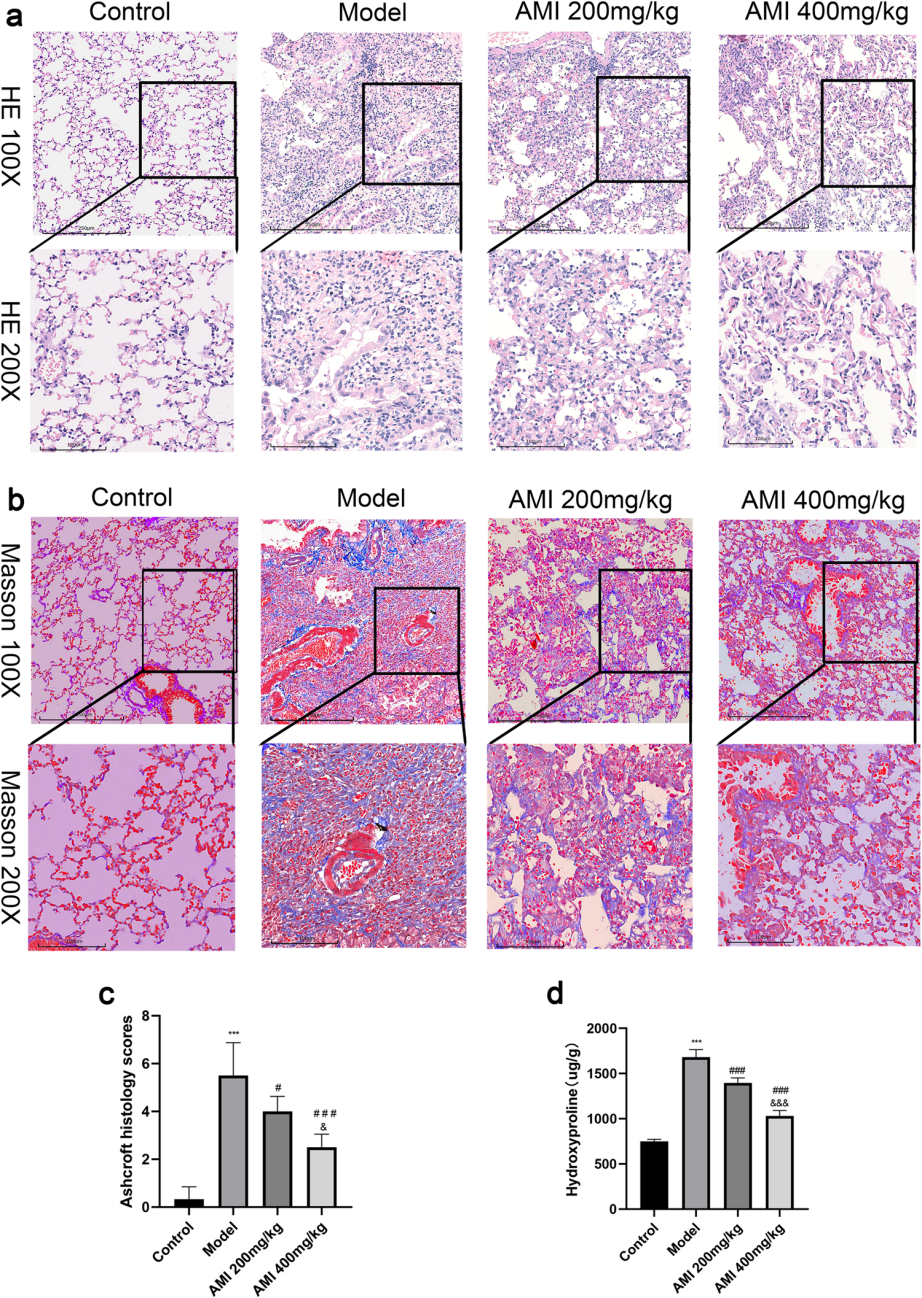
Protective effect of amifostine (AMI) on bleomycin (BLM)-induced pulmonary fibrosis in mice. (**a)** Hematoxylin and eosin staining of lung tissue sections (magnification × 100 and × 200). (**b**) Masson staining of lung tissue sections (magnification × 100 and × 200). (**c**) Ashcroft score was used to determine the degree of pulmonary fibrosis. (**d**) Content of hydroxyproline in lung tissue. Data are expressed as mean ± SD (n = 6). ^⁎⁎⁎^*P* < 0.001 versus the control group. ^###^*P* < 0.001 and ^#^*P* < 0.05 versus the model group. ^&&&^*P* < 0.001 and ^&^*P* < 0.05 versus the AMI 200-mg/kg group.

Pulmonary fibrosis was induced in mice through the use of BLM, which is a well-established model of pulmonary fibrosis. This model was then used to validate the antifibrotic effect of AMI. Hematoxylin and eosin (H&E) staining showed that the alveolar septa of mice in the control group were thinner and showed no inflammatory response, whereas the alveolar septa of mice in the model group were thickened and infiltrated by a large number of inflammatory cells. After AMI treatment, the infiltration of inflammatory cells in the lung tissue was reduced and the alveolar structure was improved (Fig. 1a). When Masson staining was performed, the lung tissue of the control group demonstrated no obvious staining, whereas the lung tissue of the model group demonstrated a large area of blue staining due to the deposition of a large number of collagen fibers in the ECM. In mice treated with AMI, the deposition of collagen fibers was decreased, leading to less blue staining in lung tissue (Fig. 1b). Mice treated with AMI also had a lower Ashcroft score than those not treated with AMI (*P* < 0.05), and a higher dose of AMI led to a greater decrease in Ashcroft score (Fig. 1c). The level of hydroxyproline is also commonly used to evaluate pulmonary fibrosis. Compared with the hydroxyproline level in the control group, the level in the model group was significantly increased (*P* < 0.05). After administration of AMI, the level of hydroxyproline decreased in a dose-dependent manner (*P* < 0.05; Fig. 1d). Overall, these findings suggest that AMI can alleviate BLM-induced lung injury and pulmonary fibrosis.

### AMI alleviates oxidative stress and inflammation in BLM-induced pulmonary fibrosis

**Figure 2:**
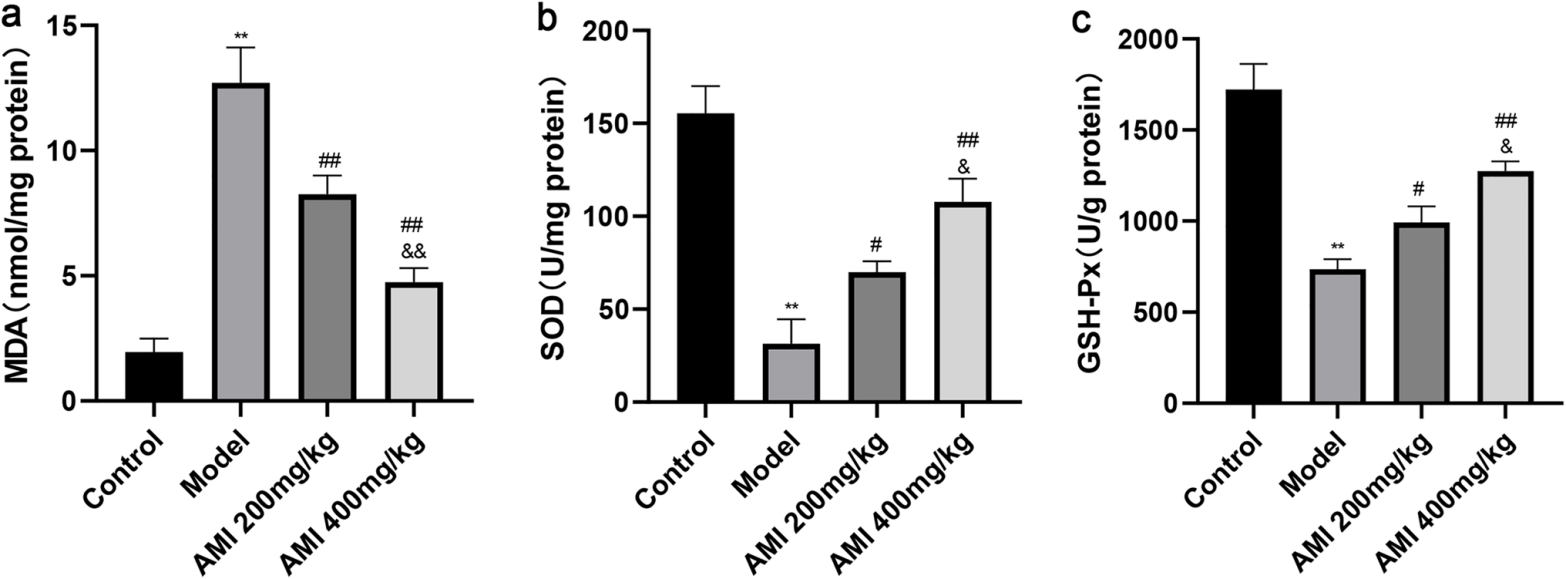
Amifostine (AMI) alleviates oxidative stress in bleomycin (BLM)-induced pulmonary fibrosis. (**a**) Lipid peroxidation malondialdehyde (MDA) levels**.** (**b**) Antioxidant enzyme superoxide dismutase (SOD) levels (**c**) Glutathione peroxidase (GSH-Px) activity in lung tissue.Data are expressed as mean ± SD (n = 6). ^⁎⁎^*P* < 0.01 versus the control group. ^##^*P* < 0.01 and ^#^*P* < 0.05 versus the model group. ^&&^*P* < 0.01 and ^&^*P* < 0.05 versus the AMI 200-mg/kg group.

**Figure 3:**
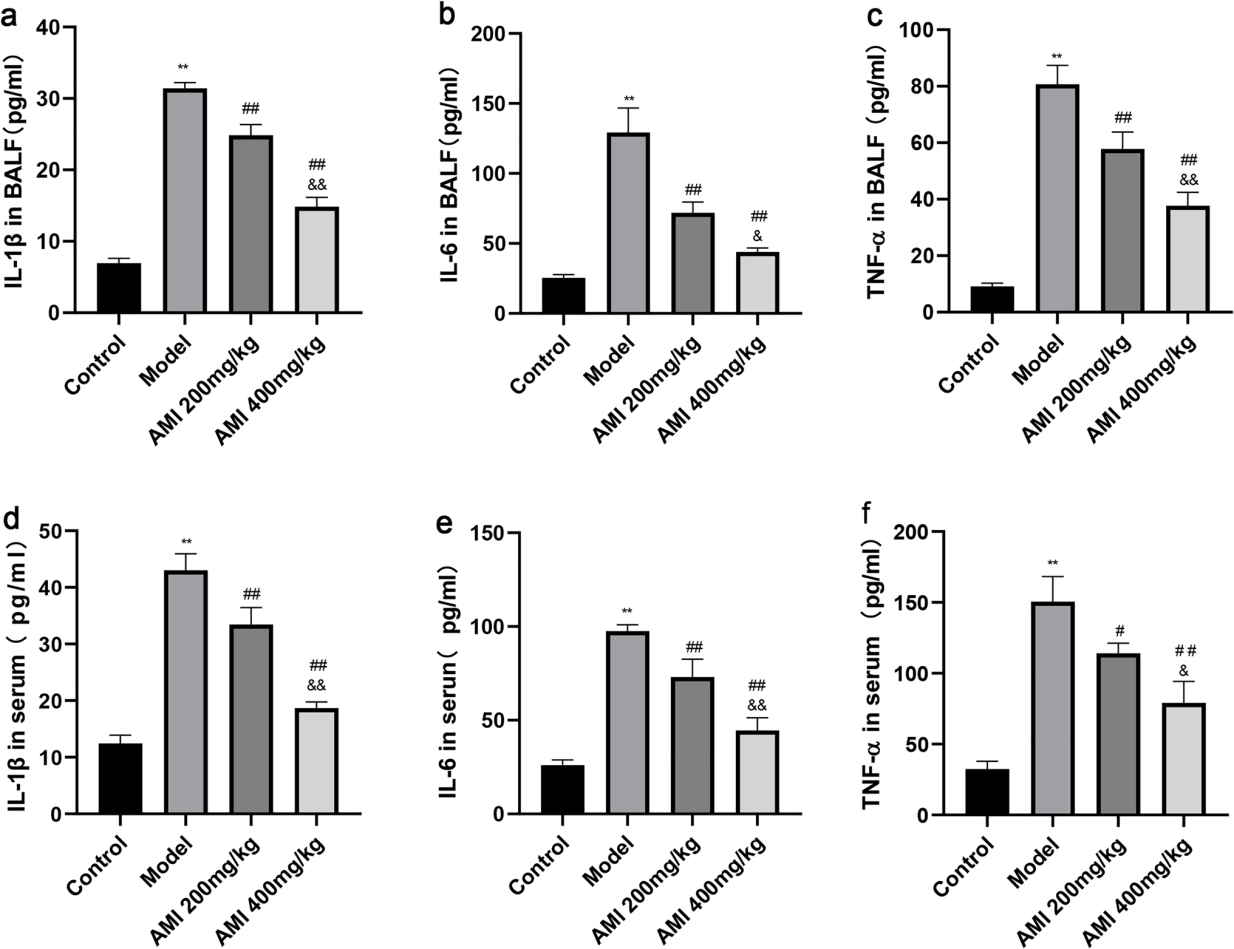
The levels of inflammatory factors IL-1β, IL-6, and TNF-α in the bronchoalveolar lavage fluid and serum were determined using enzyme-linked immunosorbent assay (ELISA) (**a**–**f**). Data are expressed as mean ± SD (n = 6). ^⁎⁎^*P* < 0.01 versus the control group. ^##^*P* < 0.01 and ^#^*P* < 0.05 versus the model group. ^&&^*P* < 0.01 and ^&^*P* < 0.05 versus the amifostine (AMI) 200-mg/kg group.

The effect of AMI on oxidative stress was evaluated by assaying the activities of malondialdehyde (MDA), superoxide dismutase (SOD), glutathione peroxidase (GSH-Px). MDA is one of the most important products of membrane lipid peroxidation, which can cause damage to the membrane system and destroy its stability. The level of MDA can therefore be used as an indicator of the degree of lipid peroxidation in tissues and organs, with a high level of MDA regarded as one of the key indicators of oxidative stress^31^. In this study, the levels of MDA in the lung tissue of the model group and the AMI treatment groups were higher than in the lung tissue of the control group. The MDA level in the AMI treatment group was lower than that in the model group, with a more obvious decrease in MDA level seen in the AMI high-dose group (*P* < 0.01; Fig. 2a). The activity of SOD and GSH-Px in the lung tissue of mice in the model group was lower than that in the control group, and increased after treatment with AMI, and the degree of difference was proportional to the dose of AMI (*P* < 0.05; Fig. 2b,c). Enzyme-linked immunosorbent assay (ELISA) demonstrated that levels of the inflammatory factors TNF-α, IL-6, and IL-1β in serum and bronchoalveolar lavage fluid of mice in the model group were significantly increased when compared with the levels in the control group, but these levels were decreased after AMI treatment in a dose-dependent fashion (*P* < 0.05; Fig. 3a–f). These results suggest that AMI may prevent BLM-induced pulmonary fibrosis by counteracting oxidative stress and inflammation.

### AMI reduces BLM-induced apoptosis of alveolar epithelial cells

**Figure 4:**
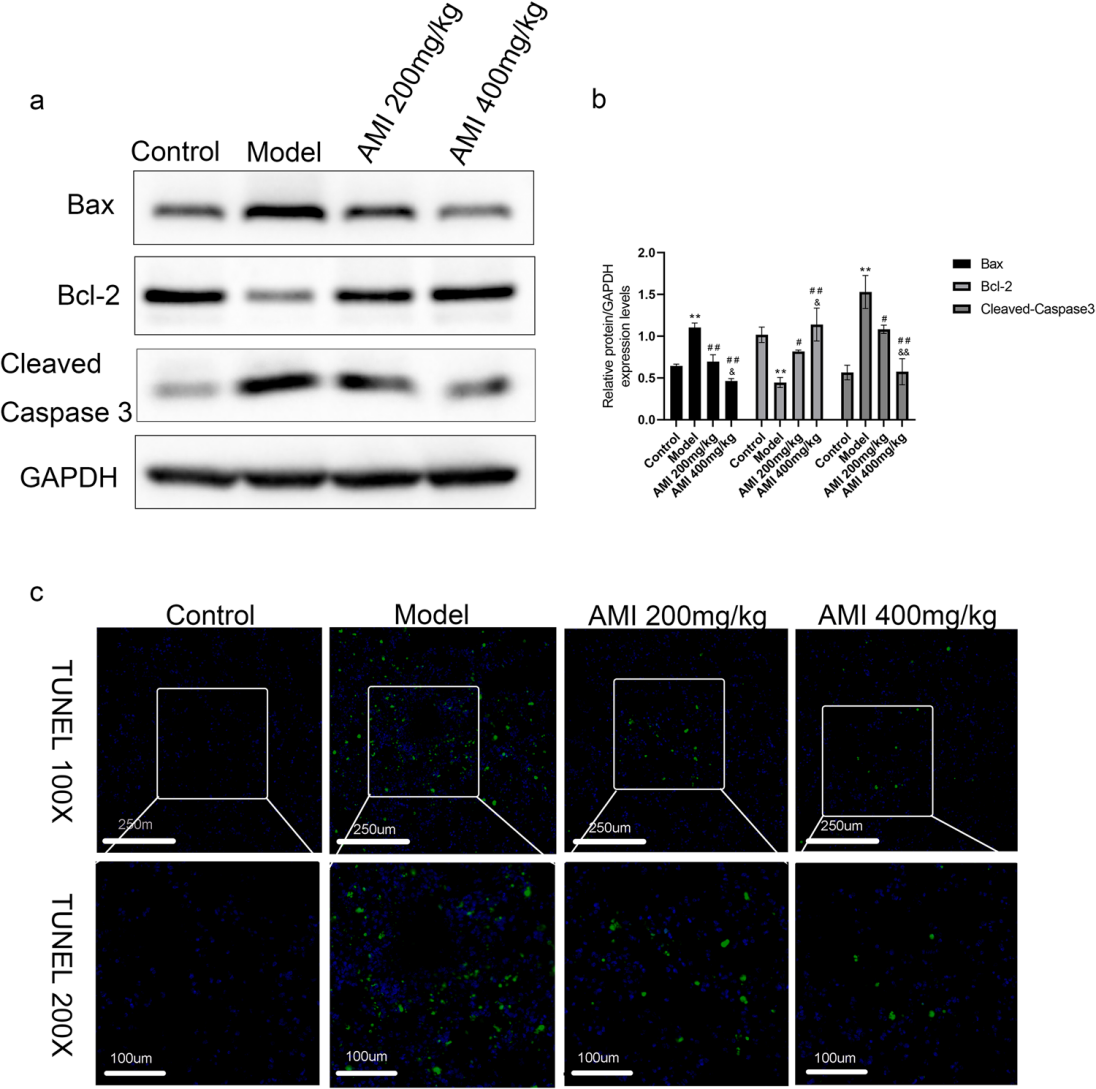
Amifostine (AMI) reduces bleomycin (BLM)-induced apoptosis of alveolar epithelial cells. (**a**) Western blot analysis was used to detect the effect of AMI on the expression of cleaved caspase-3, Bax, Bcl-2 in lung tissue. (**b**) Western blot density analysis. (**c**) Apoptotic cells in mouse lung tissue were stained with tunel kit and observed with fluorescence microscopy(magnification × 100 and × 200).Data are expressed as mean ± SD (n = 6). ^⁎⁎^*P* < 0.01 versus the control group. ^##^*P* < 0.01 and ^#^*P* < 0.05 versus the model group. ^&&^*P* < 0.01 and ^&^*P* < 0.05 versus the AMI 200-mg/kg group.

The effect of AMI on BLM-induced apoptosis of alveolar epithelial cells was investigated by measuring the expression levels of cleaved caspase-3、BAX、Bcl2 with Western blotting. Analysis of the results demonstrated that the expression level of cleaved caspase-3 and BAX in the model group was significantly higher than in the control group, and the level decreased after AMI treatment, again in a dose-dependent fashion; The expression level of Bcl2 in the model group was significantly lower than that in the control group, and the level increased in a dose-dependent manner after AMI treatment (*P* < 0.05; Fig. 4a,b). TUNEL staining of lung tissue also yielded consistent results. The number of apoptotic cells in the BLM group was significantly higher than that in the control group, and the number of apoptotic cells was significantly reduced after AMI treatment (Fig. 4c). The above results suggest that AMI could inhibit BLM-induced alveolar cell apoptosis, which may partially explain how AMI exerts a protective effect in pulmonary fibrosis.

### AMI reduces BLM-induced EMT and ECM changes in vivo

**Figure 5:**
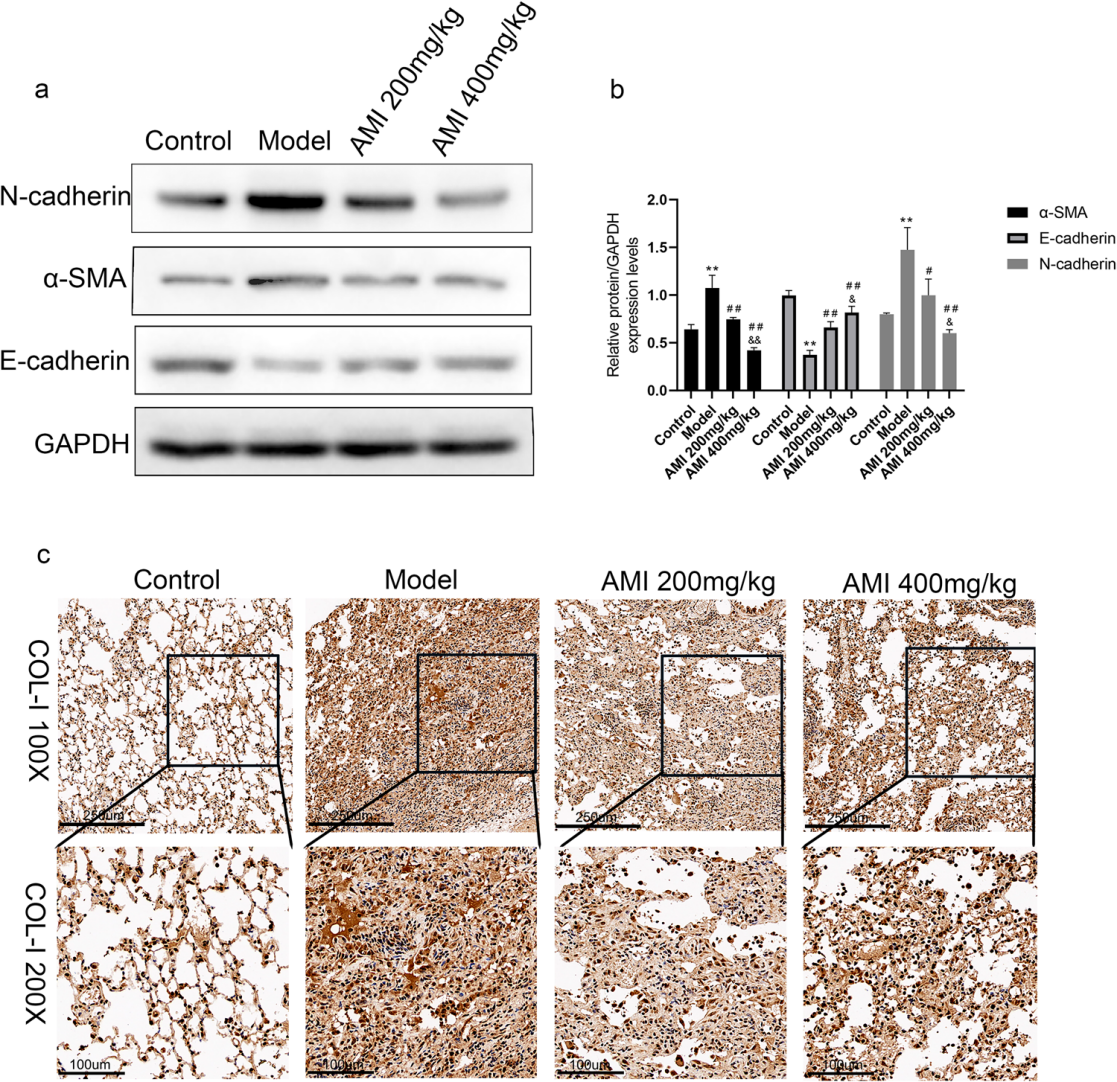
Amifostine (AMI) reduces bleomycin (BLM)-induced epithelial-mesenchymal transition (EMT) and extracellular matrix (ECM) changes. (**a**) Western blot detection results of E-cadherin, N-cadherin and α-smooth muscle actin (α-SMA) expression in lung tissue. (**b**) Western blot density analysis. (**c**) Immunohistochemical staining of type I collagen in lung tissue (magnification × 100 and × 200). Data are expressed as mean ± SD (n = 6). ^⁎⁎^*P* < 0.01 versus the control group. ^##^*P* < 0.01 and ^#^*P* < 0.05 versus the model group. ^&&^*P* < 0.01 and ^&^*P* < 0.05 versus the AMI 200-mg/kg group.

EMT is closely involved in the development of pulmonary fibrosis^32^. In this study, we used Western blotting to verify the effect of AMI on BLM-induced EMT. Compared with the control group, the level of the epithelial marker E-cadherin was decreased and the level of the mesenchymal markers N-cadherin and α-SMA were increased in the model group (*P* < 0.05; Fig. 5a,b). AMI treatment led to increased E-cadherin and decreased α-SMA and N-cadherin, with results proportional to the dose (*P* < 0.05; Fig. 5a,b). Immunohistochemical analysis demonstrated that the level of type I collagen was significantly higher in the model group than in the control group, and the level was significantly lower in the AMI treatment groups than in the model group (Fig. 5c). From these findings, it can be concluded that AMI alleviates pulmonary fibrosis by inhibiting EMT and ECM changes.

### AMI inhibits the PI3K/Akt/mTOR signaling pathway in BLM-induced pulmonary fibrosis

**Figure 6:**
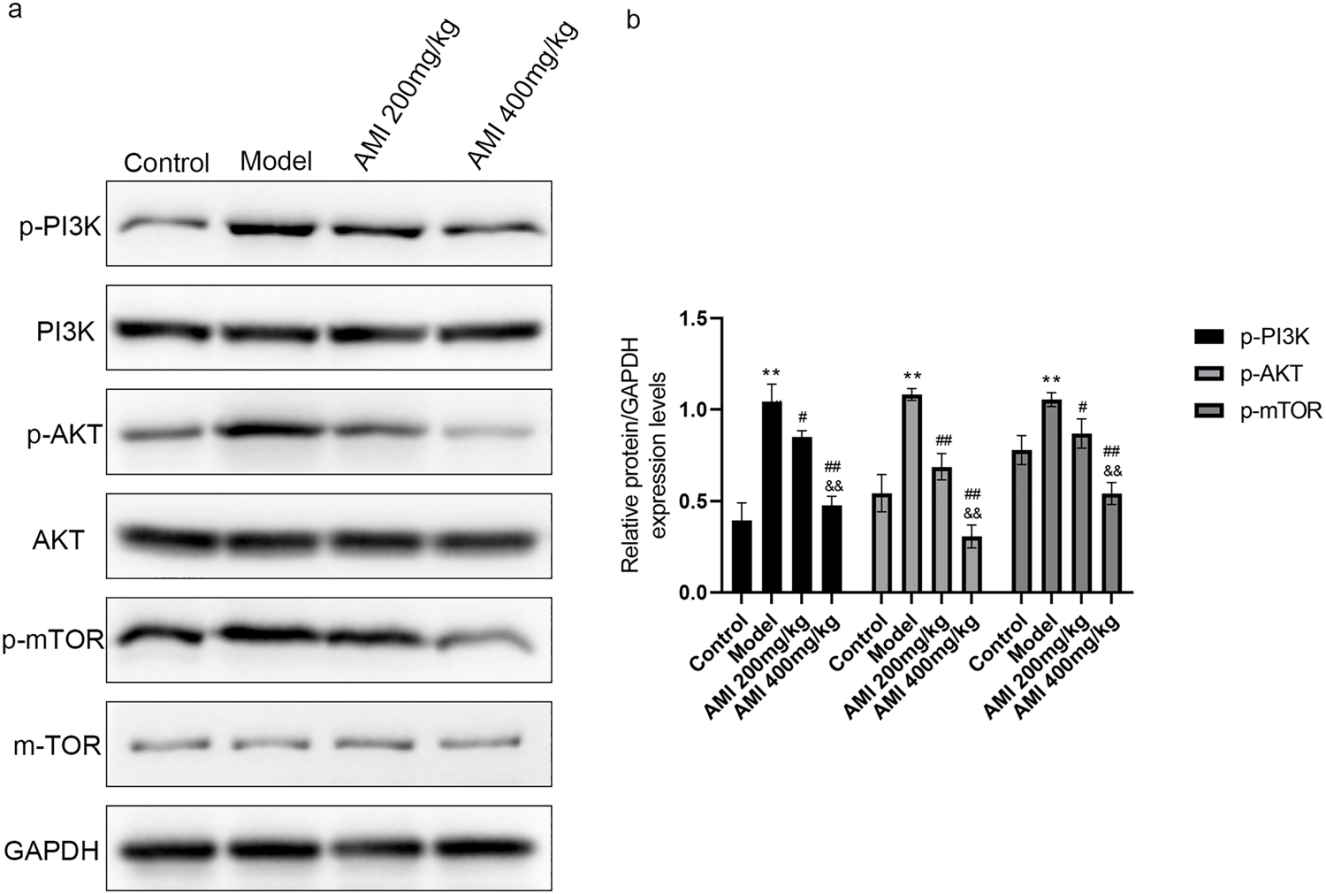
Amifostine (AMI) inhibits PI3K/Akt/mTOR signaling pathway in bleomycin (BLM)-induced pulmonary fibrosis. (**a**) The effect of AMI on p-PI3K, p-Akt, p-mTOR, PI3K, Akt, and mTOR protein expression in lung tissue was determined using Western blot analysis. (**b**) Western blot density analysis. Data are expressed as mean ± SD (n = 6). ^⁎⁎^*P* < 0.01 versus the control group. ^##^*P* < 0.01 and ^#^*P* < 0.05 versus the model group. ^&&^*P* < 0.01 versus the AMI 200-mg/kg group.

It is well known that PI3K/Akt/mTOR is an important signaling pathway involved in cell growth, survival, proliferation, apoptosis, protein synthesis, and other processes. PI3K signaling has been reported to be activated in fibrotic lung disease^33^, with animal model studies of BLM-induced pulmonary fibrosis demonstrating that inhibition of the activation of the PI3K/Akt/m-TOR signaling pathway can alleviate pulmonary fibrosis^34,35^. We explored whether the protective effect of AMI on pulmonary fibrosis can be achieved by inhibiting the PI3K/Akt/mTOR signaling pathway. The experimental results demonstrated that the phosphorylation levels of PI3K, Akt, and mTOR were significantly higher in the model group than in the control group (*P* < 0.05; Fig. 6a,b). We also found that AMI inhibited BLM-induced activation of PI3K/Akt/mTOR and reversed the elevated levels of p-PI3K, p-Akt and p-mTOR in a dose-dependent manner (*P* < 0.05). Meanwhile, the total expression of PI3K, Akt, and mTOR remained unchanged. These results suggest that AMI exerts a protective effect in pulmonary fibrosis by inhibiting the activation of PI3K/Akt/mTOR.

## Discussion

Pulmonary fibrosis comprises a heterogeneous group of diseases in which the lung parenchyma is gradually replaced by fibrotic scars, progressing to organ dysfunction and ultimately death from respiratory failure^36^. In this study, we investigated the efficacy and molecular mechanisms of AMI in a mouse model of pulmonary fibrosis. Previous research has shown that down-regulation of TGF-β/SMAD-2 by AMI inhibits EMT, reduces inflammation, and decreases collagen deposition, thus delaying pulmonary fibrosis^29^. The results of the current study are consistent with this previous research, further demonstrating that AMI alleviates pulmonary fibrosis in mice by inhibiting BLM-induced oxidative stress, inflammation, and apoptosis and by reducing EMT and ECM aggregation through inhibition of the PI3K/Akt/mTOR signaling pathway.

The progression of pulmonary fibrosis is linked to EMT, ECM aggregation, and inflammatory damage^37,38^. The EMT plays an important role in development and wound healing; however, aberrant EMT promotes the development of fibrosis^11^. Activated fibroblasts promote myofibroblast formation, and abnormal accumulation of ECM replaces normal lung tissue, resulting in structural destruction of the lung parenchyma^39,40^. Pulmonary fibrosis is therefore characterized by decreased E-cadherin levels and increased α-SMA and N-cadherin levels^41–43^. Our findings confirmed these results, and Western blotting and immunohistochemical analysis also demonstrated that AMI significantly inhibited EMT and ECM deposition.

Early in the wound healing process, an inflammatory response is generated, with inflammatory cells infiltrating the site of tissue damage^44,45^. Reactive oxygen species and profibrotic factors are produced by these activated inflammatory cells, triggering fibroblast proliferation and collagen deposition^46^. MDA, an activated carbon compound, is regarded as one of the key indicators of oxidative stress^31^ SOD, on the other hand, has the function of scavenging oxygen-free radicals and is therefore used to determine the overall status of antioxidative stress. In a previous animal model study of BLM-induced pulmonary fibrosis, the level of reactive oxygen species was significantly increased in pulmonary fibrosis, whereas GSH activity was significantly reduced, suggesting that GSH can also be used as an indicator of oxidative stress^47^. To determine whether AMI inhibits BLM-induced pulmonary fibrosis by scavenging free radicals, we measured the levels of MDA, SOD, and GSH-Px in lung tissue to assess changes in BLM-induced oxidative damage. Excessive ROS induced by oxidative stress contributes to pulmonary fibrosis by accelerating epithelial-mesenchymal transition (EMT), infiltrating of inflammatory cell and collagen accumulation^48^. We also evaluated the levels of the inflammatory cytokines TNF-α, IL-6, and IL-1β in mouse serum and bronchoalveolar lavage fluid, similar to previous studies^49,50^. We found that the activity of MDA and the levels of TNF-α, IL-6, and IL-1β were significantly decreased in the AMI treatment group, and the activities of SOD and GSH-Px were increased in a dose-dependent manner. These results suggest that AMI may prevent BLM-induced pulmonary fibrosis by counteracting oxidative stress and inflammation.

Oxidative stress plays another role in pulmonary fibrosis by causing activation of the PI3K/Akt pathway^51^. Previous research has shown that PI3K/Akt signaling is closely involved in pulmonary fibrosis development^16,52,53^. Activation of this pathway promotes increased expression of α-SMA and EMT, accelerating fibrosis^13,54^. The interaction between the PI3K/Akt signaling pathway and TGF-β has also been found to promote the occurrence and development of pulmonary fibrosis^14^. Furthermore, activation of PI3K/Akt promotes activation of the downstream factor mTOR, which is an autophagy regulator^15,16^. PI3K/Akt/mTOR activation reduces autophagy and increases apoptosis of alveolar epithelial cells, thus promoting pulmonary fibrosis^55^. In this study, we therefore assessed the expression of PI3K/Akt/mTOR signaling pathway-related proteins in AMI-treated mice. Western blot analysis demonstrated that the phosphorylation levels of PI3K, Akt, and mTOR in the model group were significantly higher than in the control group; after AMI treatment, the protein contents of phosphorylated PI3K, phosphorylated Akt, and phosphorylated mTOR were significantly decreased. This study is the first to show that the effect of AMI in pulmonary fibrosis is at least partially achieved through inhibition of the PI3K/Akt/mTOR signaling pathway.

Finally, the pathogenesis of various lung diseases is also closely related to apoptosis^56^. Repeated injury of alveolar epithelial cells causes apoptosis, which is followed by abnormal lung repair and fibroblast activation, leading to progressive fibrosis^57^. Bcl-2 can alleviate cell apoptosis by decreasing ROS generation, while Bax can inhibit the expression of Bcl-2 as a pro-apoptotic factor ^58^. The pro-apoptotic Bcl-2 family members Bax can lead to the mitochondrial membrane permeability and cytochrome c release, which all induce the caspase activation and cell death^59^. Epletion of GSH results in a redox imbalance and increases caspase-3 levels^60^, and research has shown that BLM-induced oxidative stress leads to direct apoptosis of alveolar epithelial cells through the activation of caspases^61^. Apoptosis inhibitors are therefore widely used to treat lung injury and early fibrosis. Plataki et al. found that Bax expression increased and Bcl-2 expression decreased in epithelial cells of patients with IPF^62^.

In this study, we found that the expression of caspase-3 and Bax in the model group increased, and the expression of Bcl-2 decreased, which were all reversed by AMI treatment in a dose-dependent manner,suggesting that AMI can inhibit alveolar epithelial cell apoptosis in pulmonary fibrosis.

A limitation of this study is that only two concentration gradients were set for AMI, and we were unable to assess whether higher doses of AMI would produce different results. Therefore, this issue needs to be further clarified in subsequent studies.

In conclusion, our study demonstrates that AMI alleviates pulmonary fibrosis in mice by inhibiting BLM-induced oxidative stress, inflammation, and apoptosis and by reducing EMT and ECM aggregation through inhibition of the PI3K/Akt/mTOR signaling pathway. These findings suggest that AMI has potential as a multitarget agent for the treatment of pulmonary fibrosis.

## Methods

### Animals and experimental design

This animal experiment was carried out in strict accordance with the Guidelines for the Care and Use of Laboratory Animals issued by the National Institutes of Health and the regulations of the Animal Ethics Experiment Committee of Guizhou Medical University (Approval number: Qian 2,101,424). This study was also carried out in compliance with the ARRIVE guidelines^63^. Six- to eight-week-old male C57BL/6 J mice were obtained from Changsha Tianqin Biotechnology (Hunan, China). (Experimental Animal Certificate No.: SCXK (Xiang) 2019–0014). A total of 48 mice weighing between 20 and 25 g were used in the experiments.The mice were housed under controlled temperature (22–26 °C) and a 12-h light–dark cycle. The mice had free access to food and water. Based on the principle of random allocation, the mice were divided into 4 groups with 12 mice in each group: a control group, a model group, a 200-mg/kg AMI group, and a 400-mg/kg AMI group. Dose selection is based on previous studies^25,64,65^.

Intratracheal BLM administration is as previously described^66^. In short, the mice were anesthetized by intraperitoneal injection of 1% pentobarbital sodium, and then injected BLM or normal saline into the trachea with sterile insulin syringe. After injection, the animals were immediately upright and rotated left and right to make the drug solution evenly distributed in the lungs.Mice in model group and AMI group were injected BLM into trachea (5 mg/kg, MedChemExpress, HY-17565A); mice in the control group were injected with the same volume of normal saline. The mice in the control group and model group were intraperitoneally injected 200 ul sterile saline per day from day 2–21 after BLM injection, while the mice in the 200 mg/kg AMI group were intraperitoneally injected with 200 mg/kg AMI (MedChemExpress, HY-B0639) every day from day 2–21 after BLM injectio, and the mice in the 400 mg/kg AMI group were intraperitoneally injected with 400 mg/kg AMI every day from day 2–21 after BLM injection. According to the requirements of the American Veterinary Association’s Euthanasia Guidelines 2020, Mice were euthanized on day 21 via an excessive intraperitoneal injection of pentobarbital sodium, blood and bronchoalveolar lavage fluid were quickly collected, and the lungs were separated. Fix a portion of lung tissue with 4% paraformaldehyde and embed in paraffin 5 μm sections were used for Hematoxylin and Eosin (HE), Masson, TUNEL and Immunohistochemical staining, and the remaining lung tissue was stored at − 80° for subsequent experiments. Mice within each group were then analyzed in a blinded fashion.

### H&E staining

Morphological changes in the lungs were assessed using H&E staining. The lung tissue was fixed with 4% paraformaldehyde and embedded in paraffin for sectioning. Paraffin sections were then stained with H&E to identify histopathological changes. The degree of pulmonary fibrosis was expressed using the Ashcroft score^67^. To obtain this score, 30 fields of view were freely selected for each section under a microscope (Olympus, Tokyo, Japan) so that the degree of fibrosis in H&E-stained tissue sections could be semiquantitatively analyzed. The degree of tissue fibrosis was given a score ranging from 0 to 8, and the mean of all visual field scores was used as the fibrosis degree score for the sample. Three pathologists were invited to perform this evaluation, and they were blinded to prevent observer bias. The specific criteria for scoring were as follows: 0 points, normal lung tissue; 1 point, mild fibrosis of bronchioles and alveolar walls; 3 points, moderate fibrosis of bronchioles and alveolar walls but no obvious damage to lung tissue structure; 5 points, obvious fibrosis with destruction of lung tissue structure and formation of fibrous strips or small fibrous nodules; 7 points, severe lung tissue structural deformation or large-area fibrous foci; and 8 points, fibrotic lesions in the entire visual field. 2 points, microscopic appearance between 1 point Between 3 points; 4 points, microscopic appearance between 3 and 5 points; 6 points, microscopic appearance between 5 and 7 points.

### Masson’s trichrome staining

Masson’s trichrome staining evaluates pulmonary interstitial fibrosis as previously described^68^. The lung tissue was isolated, embedded in paraffin, and sectioned. Sections were then stained using Masson’s tricolor kit according to the manufacturer’s instructions (Solarbio, Beijing, China). This allowed us to identify pathological changes in lung tissue, which were then photographed under a light microscope (Olympus).

### Biochemical analysis

Once the mice were anesthetized, blood was collected from the eyeball and placed into a centrifuge tube. The tube was left at room temperature for 30 min and was then centrifuged (2500×*g*) at 4 °C for 10 min. After centrifugation, the supernatant was stored at − 80 °C. Bronchoalveolar lavage fluid was collected through tracheal intubation and washed 3 times with 300 μL of normal saline each time. The recovered lavage fluid was centrifuged (2500×*g*) at 4 °C for 10 min, and the supernatant was removed and stored at − 80 °C for subsequent experiments. Serum and bronchoalveolar lavage fluid were then analyzed for TNF-α (CME0004, Sizhengbai Biotechnology Co., Ltd., Beijing, China), IL-6 (CME0006, Sizhengbai Biotechnology Co., Ltd.), and IL-1β (CME0015, Sizhengbai Biotechnology Co., Ltd.) levels using ELISA detection kits according to the manufacturer’s instructions. The hydroxyproline content in lung homogenates was examined using a hydroxyproline detection kit (Nanjing Jiancheng Bioengineering Institute, Jiangsu, China) according to the manufacturer’s protocol, and the optical density at 550 nm was determined on a spectrophotometer. In addition, lung tissue MDA (catalog number: A003-1), superoxide dismutase (SOD, catalog number: A001-3) and glutathione peroxidase (GSH-PX, catalog number: A005-1) activities were measured using the corresponding assay kits(Nanjing Jiancheng Biotechnology Research Institute, Jiangsu, China) according to the manufacturer's protocol.

### Terminal dUTP nick-end labeling (TUNEL)

In the early stage of apoptosis, nuclear DNA is fragmented, and Tunel staining is an experimental method to identify apoptotic cells by targeting the fragmented DNA.Take the paraffin section of lung tissue, put the section in fresh xylene for dewaxing, hydrate the section with absolute ethanol, 90% ethanol, 70% ethanol and distilled water in turn, add proteinase K solution to incubate, and prepare Tunel detection solution. In dark conditions, add Tunel detection solution to make the staining solution fully cover the sample, and at the same time add a coverslip to prevent the dye solution from volatilizing, incubate at 37 °C for 1 h for staining, wash the dye solution with PBS, and then add DAPI to stain the nucleus, and then mount the tissue stains with an anti-fluorescence quencher. Under a fluorescence microscope, the green fluorescence intensity was observed and photographed.

### Immunohistochemical staining

Lung tissue was embedded in paraffin blocks, sectioned, dried, deparaffinized, hydrated, and then subjected to antigen retrieval. Lung tissue sections were incubated overnight at 4 °C with anti-collagen type I (1:100; Cell Signaling Technology, USA) primary antibody. The sections were then washed 3 times with phosphate buffered saline, and working droplets were prepared on tissue sections using a DAB immunohistochemical staining kit. Type I collagen expression and distribution were then observed under a light microscope (Olympus).

### Western blotting

Western blotting was used to detect α-SMA (1:1000, Cell Signaling Technology), E-cadherin (1:10,000, Abcam,UK), cleaved caspase-3 (1:1000, Cell Signaling Technology), p-PI3K (1:1000, Abcam), p-Akt (1:2000, Cell Signaling Technology), p-mTOR (1:1000, Cell Signaling Technology), PI3K (1:1000, Cell Signaling Technology), Akt (1:1000, Cell Signaling Technology), mTOR (1:1000, Cell Signaling Technology), and GAPDH (1:10,000, Abcam) protein levels. Using RIPA buffer (Solarbio) to add protease inhibitors (Solarbio) and phosphatase inhibitors (Solarbio), the lung tissue was thoroughly ground with a grinders and centrifuged (12,000 rpm) at 4 °C for 10 min, and the supernatant was removed for subsequent experiments. Total protein concentration was calculated using the BCA protein detection kit (Pierce, Rockford, IL, USA). A total of 10 μg of protein was added to each channel of an SDS–polyacrylamide gel. The proteins were electrophoretically separated into different layers and transferred to PVDF membranes (Immobilon-P, Darmstadt, Germany) using a wet transfer system.Western blots were cut prior to hybridisation with antibodies during blotting.TBST buffer, and incubated with primary antibody overnight at 4 °C. The primary antibody was then recovered, washed with TBST buffer, and added with a secondary antibody (IgG, 1:5000, Cell Signaling Technology)incubated at room temperature for 1.5 h, washed with TBST buffer, and finally developed with ECL (Thermo Fisher Scientific, Waltham, MA, USA) exposure solution. Grayscale values were then analyzed with ImageJ software.

## Experiment end day. December 25, 2022

### Statistical analysis

The data are presented as mean ± SD. One-way ANOVA was used for comparisons between experimental groups. For multiple testing, Bonferroni analysis was performed. *P* values < 0.05 were considered statistically significant.

## Supplementary Information

Supplementary Information 1.

Supplementary Information 2.

Supplementary Information 3.

Supplementary Information 4.

Supplementary Information 5.

Supplementary Information 6.

Supplementary Information 7.

Supplementary Information 8.

Supplementary Information 9.

Supplementary Information 10.

Supplementary Information 11.

Supplementary Information 12.

## Data Availability

The datasets generated during and/or analyzed during the current study are available from the corresponding author upon reasonable request.

## References

[1] Meduri GU, Eltorky MA (2015). Understanding ARDS-associated fibroproliferation. Intensive Care Med..

[2] Vancheri C (2018). Nintedanib with add-on pirfenidone in idiopathic pulmonary fibrosis. Results of the INJOURNEY trial. Am. J. Respir. Crit. Care Med..

[3] Selman M (2001). Idiopathic pulmonary fibrosis: Prevailing and evolving hypotheses about its pathogenesis and implications for therapy. Ann. Intern. Med..

[4] Bellaye PS, Kolb M (2015). Why do patients get idiopathic pulmonary fibrosis? Current concepts in the pathogenesis of pulmonary fibrosis. BMC Med..

[5] Maher TM, Wells AU, Laurent GJ (2007). Idiopathic pulmonary fibrosis/multiple causes and multiple mechanisms?. Eur. Respir. J..

[6] Liu YM, Nepali K, Liou JP (2017). Idiopathic pulmonary fibrosis: Current status, recent progress, and emerging targets. J. Med. Chem..

[7] Kolb M, Bonella F, Wollin L (2017). Therapeutic targets in idiopathic pulmonary fibrosis. Respir. Med..

[8] Hung C (2013). Role of lung pericytes and resident fibroblasts in the pathogenesis of pulmonary fibrosis. Am. J. Respir. Crit. Care Med..

[9] Luppi F, Kalluri M, Faverio P, Kreuter M, Ferrara G (2021). Idiopathic pulmonary fibrosis beyond the lung: Understanding disease mechanisms to improve diagnosis and management. Respir. Res..

[10] Cruz-Solbes AS, Youker K (2017). Epithelial to mesenchymal transition (EMT) and endothelial to mesenchymal transition (EndMT): Role and implications in kidney fibrosis. Results Probl. Cell Differ..

[11] Lamouille S, Xu J, Derynck R (2014). Molecular mechanisms of epithelial-mesenchymal transition. Nat. Rev. Mol. Cell Biol..

[12] Yan WW (2017). MiR-503 modulates epithelial-mesenchymal transition in silica-induced pulmonary fibrosis by targeting PI3K p85 and is sponged by lncRNA MALAT1. Sci. Rep..

[13] Conte E (2011). Inhibition of PI3K prevents the proliferation and differentiation of human lung fibroblasts into myofibroblasts: the role of class I P110 isoforms. PLoS ONE.

[14] Sun Y, Zhang Y, Chi P (2018). Pirfenidone suppresses TGFbeta1induced human intestinal fibroblasts activities by regulating proliferation and apoptosis via the inhibition of the Smad and PI3K/AKT signaling pathway. Mol. Med. Rep..

[15] Conte E (2013). PI3K p110gamma overexpression in idiopathic pulmonary fibrosis lung tissue and fibroblast cells: In vitro effects of its inhibition. Lab. Invest..

[16] Nie Y (2017). AKT2 regulates pulmonary inflammation and fibrosis via modulating macrophage activation. J. Immunol..

[17] Wang J (2022). Targeting PI3K/AKT signaling for treatment of idiopathic pulmonary fibrosis. Acta Pharm. Sin. B..

[18] Arora A (2018). Amifostine analog, DRDE-30, attenuates bleomycin-induced pulmonary fibrosis in mice. Front. Pharmacol..

[19] Arora A, Bhuria V, Singh S (2022). Amifostine analog, DRDE-30, alleviates radiation induced lung damage by attenuating inflammation and fibrosis. Life Sci..

[20] Arora A, Bhuria V, Hazari PP (2018). Amifostine analog, DRDE-30, attenuates bleomycin-induced pulmonary fibrosis in mice. Front Pharmacol..

[21] Travis EL, Newman RA, Helbing SJ (1987). WR 2721 modification of type II cell and endothelial cell function in mouse lung after single doses of radiation. Int. J. Radiat. Oncol. Biol. Phys..

[22] Vujaskovic Z, Feng QF, Rabbani ZN, Samulski TV, Anscher MS, Brizel DM (2002). Assessment of the protective effect of amifostine on radiation-induced pulmonary toxicity. Exp. Lung Res..

[23] Antonadou D (2003). Amifostine reduces radiochemotherapy-induced toxicities in patients with locally advanced non-small cell lung cancer. Semin. Oncol..

[24] Sasse AD, Sasse EC, Clark OAC, de Oliveira Clark LG (2006). Amifostine reduces side effects and improves complete response rate during radiotherapy: Results of a meta-analysis. Int. J. Radiat. Oncol. Biol. Phys..

[25] Wang Y, Dai W, Li W (2019). Protective effect and mechanism of amifotin in pulmonary fibrosis rats. PLA Med. J..

[26] Devine A, Marignol L (2016). Potential of amifostine for chemoradiotherapy and radiotherapy-associated toxicity reduction in advanced NSCLC: A meta-analysis. Anticancer Res..

[27] Komaki R (2004). Effects of amifostine on acute toxicity from concurrent chemotherapy and radiotherapy for inoperable non-small-cell lung cancer: report of a randomized comparative trial. Int. J. Radiat. Oncol. Biol. Phys..

[28] Antonadou D (2001). Randomized phase III trial of radiation treatment +/− amifostine in patients with advanced-stage lung cancer. Int. J. Radiat. Oncol. Biol. Phys..

[29] Dadrich M (2015). Combined inhibition of TGFbeta and PDGF signaling attenuates radiation-induced pulmonary fibrosis. Oncoimmunology.

[30] Yuan, W. Protective effect and mechanism of amifostine on bleomycin-induced pulmonary fibrosis in mice. Chengdu Medical College Master Degree Thesis. (2020).

[31] Lei Y (2015). Redox regulation of inflammation: Old elements, a new story. Med. Res. Rev..

[32] Salton F, Volpe MC, Confalonieri M (2019). Epithelial-mesenchymal transition in the pathogenesis of idiopathic pulmonary fibrosis. Medicina.

[33] Mercer PF (2016). Exploration of a potent PI3 kinase/mTOR inhibitor as a novel anti-fibrotic agent in IPF. Thorax.

[34] Huang C (2016). Kca3.1 mediates dysfunction of tubular autophagy in diabetic kidneys via PI3k/Akt/mTOR signaling pathways. Sci. Rep..

[35] Chitra P, Saiprasad G, Manikandan R, Sudhandiran G (2015). Berberine inhibits Smad and non-Smad signaling cascades and enhances autophagy against pulmonary fibrosis. J. Mol. Med. (Berl.).

[36] Wynn TA (2011). Integrating mechanisms of pulmonary fibrosis. J. Exp. Med..

[37] Wolters PJ, Collard HR, Jones KD (2014). Pathogenesis of idiopathic pulmonary fibrosis. Annu. Rev. Pathol..

[38] Li L (2015). Total extract of Yupingfeng attenuates bleomycin-induced pulmonary fibrosis in rats. Phytomedicine.

[39] Phan THG (2021). Emerging cellular and molecular determinants of idiopathic pulmonary fibrosis. Cell. Mol. Life Sci..

[40] Hewlett JC, Kropski JA, Blackwell TS (2018). Idiopathic pulmonary fibrosis: Epithelial-mesenchymal interactions and emerging therapeutic targets. Matrix Biol..

[41] Cannito S (2010). Epithelial-mesenchymal transition: From molecular mechanisms, redox regulation to implications in human health and disease. Antioxid. Redox Signal..

[42] Tanjore H (2009). Contribution of epithelial-derived fibroblasts to bleomycin-induced lung fibrosis. Am. J. Respir. Crit. Care Med..

[43] Lin HY (2018). Notch3 inhibits epithelial-mesenchymal transition in breast cancer via a novel mechanism, upregulation of GATA-3 expression. Oncogenesis.

[44] Eming SA, Wynn TA, Martin P (2017). Inflammation and metabolism in tissue repair and regeneration. Science.

[45] Smigiel KS, Parks WC (2018). Macrophages, wound healing, and fibrosis: Recent insights. Curr. Rheumatol. Rep..

[46] Barron L, Wynn TA (2011). Fibrosis is regulated by Th2 and Th17 responses and by dynamic interactions between fibroblasts and macrophages. Am. J. Physiol. Gastrointest. Liver Physiol..

[47] Serrano-Mollar A (2003). In vivo antioxidant treatment protects against bleomycin-induced lung damage in rats. Br. J. Pharmacol..

[48] Cheresh P, Kim SJ, Tulasiram S, Kamp DW (2013). Oxidative stress and pulmonary fibrosis. Biochim. Biophys. Acta..

[49] Yu WN, Sun LF, Yang H (2016). Inhibitory effects of astragaloside IV on bleomycin- induced pulmonary fibrosis in rats via attenuation of oxidative stress and inflammation. Inflammation.

[50] Ermis H (2013). Protective effect of dexpanthenol on bleomycin-induced pulmonary fibrosis in rats. Naunyn Schmiedebergs Arch. Pharmacol..

[51] Lee KI (2020). Ultrafine silicon dioxide nanoparticles cause lung epithelial cells apoptosis via oxidative stress-activated PI3K/Akt-mediated mitochondria- and endoplasmic reticulum stress-dependent signaling pathways. Sci. Rep..

[52] Zhai C (2014). Selective inhibition of PI3K/Akt/mTOR signaling pathway regulates autophagy of macrophage and vulnerability of atherosclerotic plaque. PLoS ONE.

[53] Larson-Casey JL, Deshane JS, Ryan AJ, Thannickal VJ, Carter AB (2016). Macrophage Akt1 kinase-mediated mitophagy modulates apoptosis resistance and pulmonary fibrosis. Immunity.

[54] Zhang XL, Xing RG, Chen L, Liu CR, Miao ZG (2016). PI3K/Akt signaling is involved in the pathogenesis of bleomycin-induced pulmonary fibrosis via regulation of epithelial-mesenchymal transition. Mol. Med. Rep..

[55] Liu MW (2019). Ligustrazin increases lung cell autophagy and ameliorates paraquat-induced pulmonary fibrosis by inhibiting PI3K/Akt/mTOR and hedgehog signalling via increasing miR-193a expression. BMC Pulm. Med..

[56] Fattman CL (2008). Apoptosis in pulmonary fibrosis: too much or not enough?. Antioxid. Redox Signal..

[57] Uhal BD (2008). The role of apoptosis in pulmonary fibrosis. Eur. Respir. Rev..

[58] Yu W, Sun H, Zha W, Cui W, Xu L, Min Q, Wu J (2017). Apigenin attenuates adriamycin-induced cardiomyocyte apoptosis via the PI3K/AKT/mTOR pathway. Evid. Based. Complement. Alternat. Med..

[59] Gordy C, He YW (2012). The crosstalk between autophagy and apoptosis: Where does this lead?. Prot. Cell.

[60] Ma X (2019). Protective effects of functional amino acids on apoptosis, inflammatory response, and pulmonary fibrosis in lipopolysaccharide-challenged mice. J. Agric. Food Chem..

[61] Wallach-Dayan SB (2006). Bleomycin initiates apoptosis of lung epithelial cells by ROS but not by Fas/FasL pathway. Am. J. Physiol. Lung Cell. Mol. Physiol..

[62] Plataki M, Koutsopoulos AV, Darivianaki K, Delides G, Siafakas NM, Bouros D (2005). Expression of apoptotic and antiapoptotic markers in epithelial cells in idiopathic pulmonary fibrosis. Chest J..

[63] PercieduSert N (2020). Reporting animal research: Explanation and elaboration for the ARRIVE guidelines 20. PLOS Biol..

[64] Su L, Huang S, Yang G (2014). Experimental study of amifortine on the protection of radiation lung injury in mice. China J. Modern Med..

[65] Xuan W, Li S, Wu X (2018). Protective effect of amifotin on radiation lung injury in mice. China J. Modern Med..

[66] Jiang D, Liang J, Hodge J, Lu B, Zhu Z, Yu S (2004). Regulation of pulmonary fibrosis by chemokine receptor CXCR3. J. Clin. Invest..

[67] Ashcroft T, Simpson JM, Timbrell V (1988). Simple method of estimating severity of pulmonary fibrosis on a numerical scale. J. Clin. Pathol..

[68] Li X, Li C, Tang Y, Huang Y, Cheng Q, Huang X, Zhao F, Hao C, Feng D, Xu J, Han J, Tang S, Liu W, Yue S, Luo Z (2018). NMDA receptor activation inhibits the antifibrotic effect of BM-MSCs on bleomycin-induced pulmonary fibrosis. Am. J. Physiol.-Lung Cell. Mol. Physiol..

