# Supplementary material for: Amifostine attenuates bleomycin-induced pulmonary fibrosis in mice through inhibition of the PI3K/Akt/mTOR signaling pathway
Source: Sci Rep. 2023 Jun 28;13:10485. doi: 10.1038/s41598-023-34060-8 (PMC10307827; doi:10.1038/s41598-023-34060-8)
Supplement: Supplementary file 1 — Supplementary Information 1. [file 41598_2023_34060_MOESM1_ESM.docx]

| 1 Bax |  |  |
| --- | --- | --- |
| 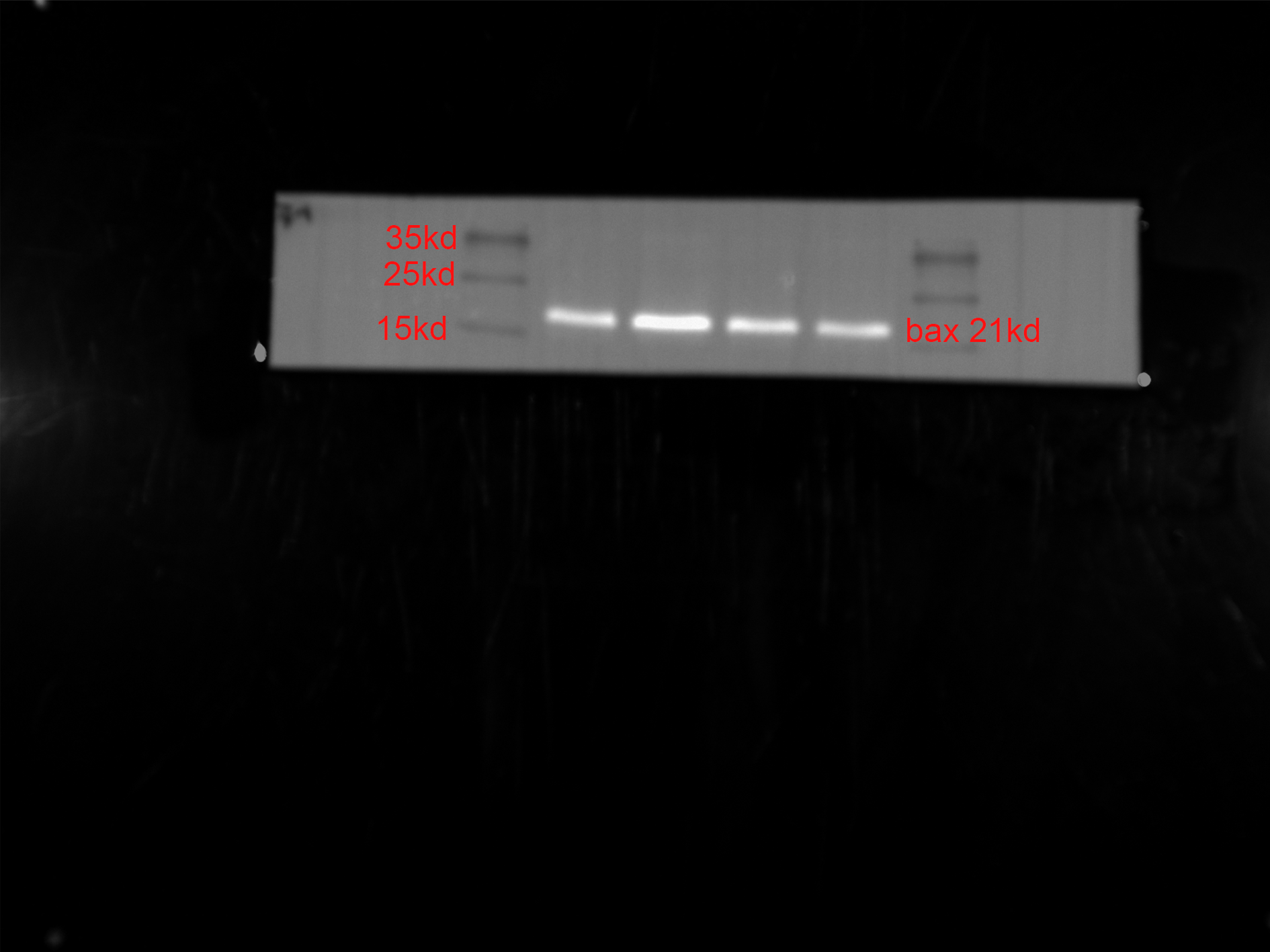 | 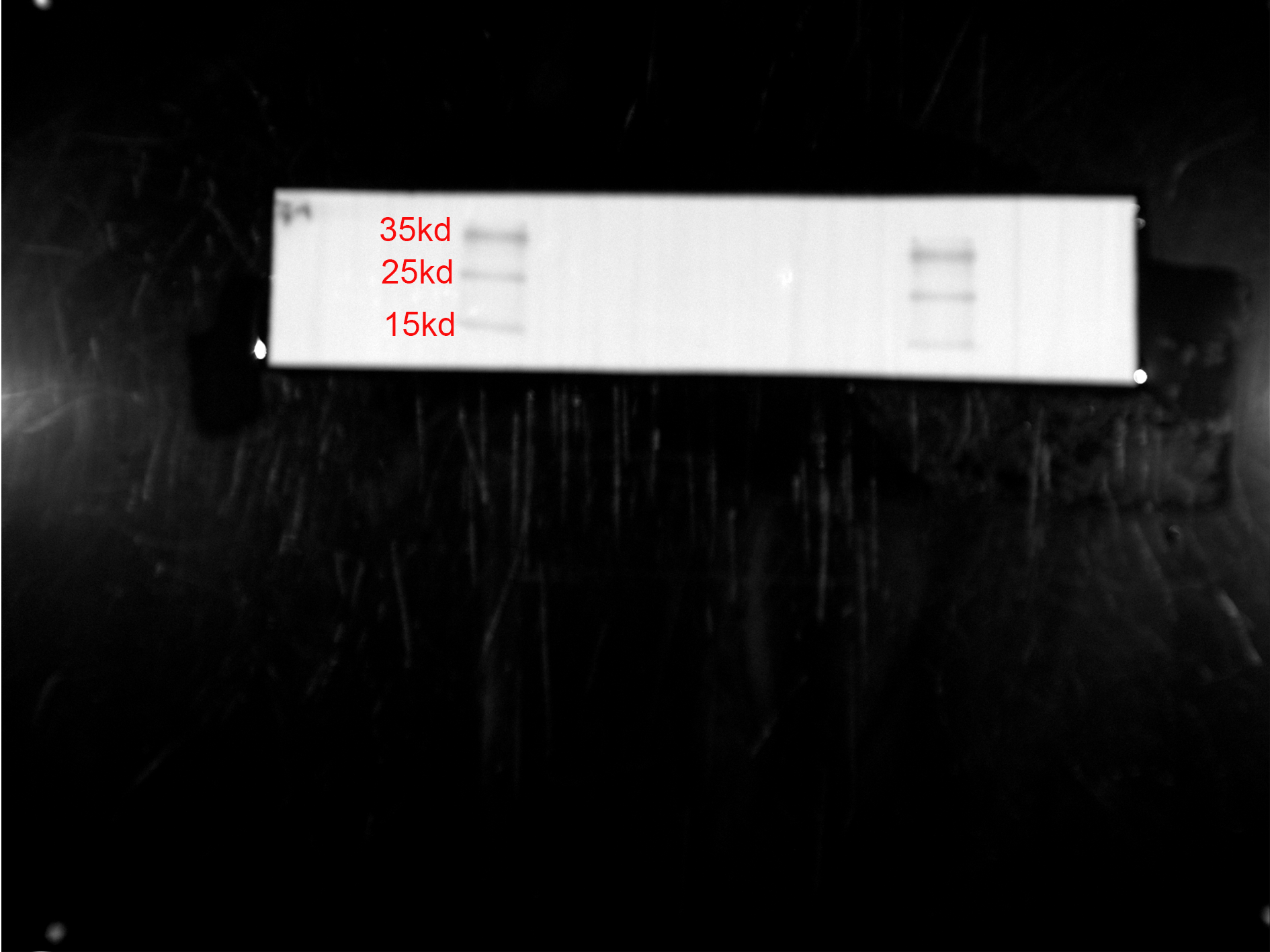 | 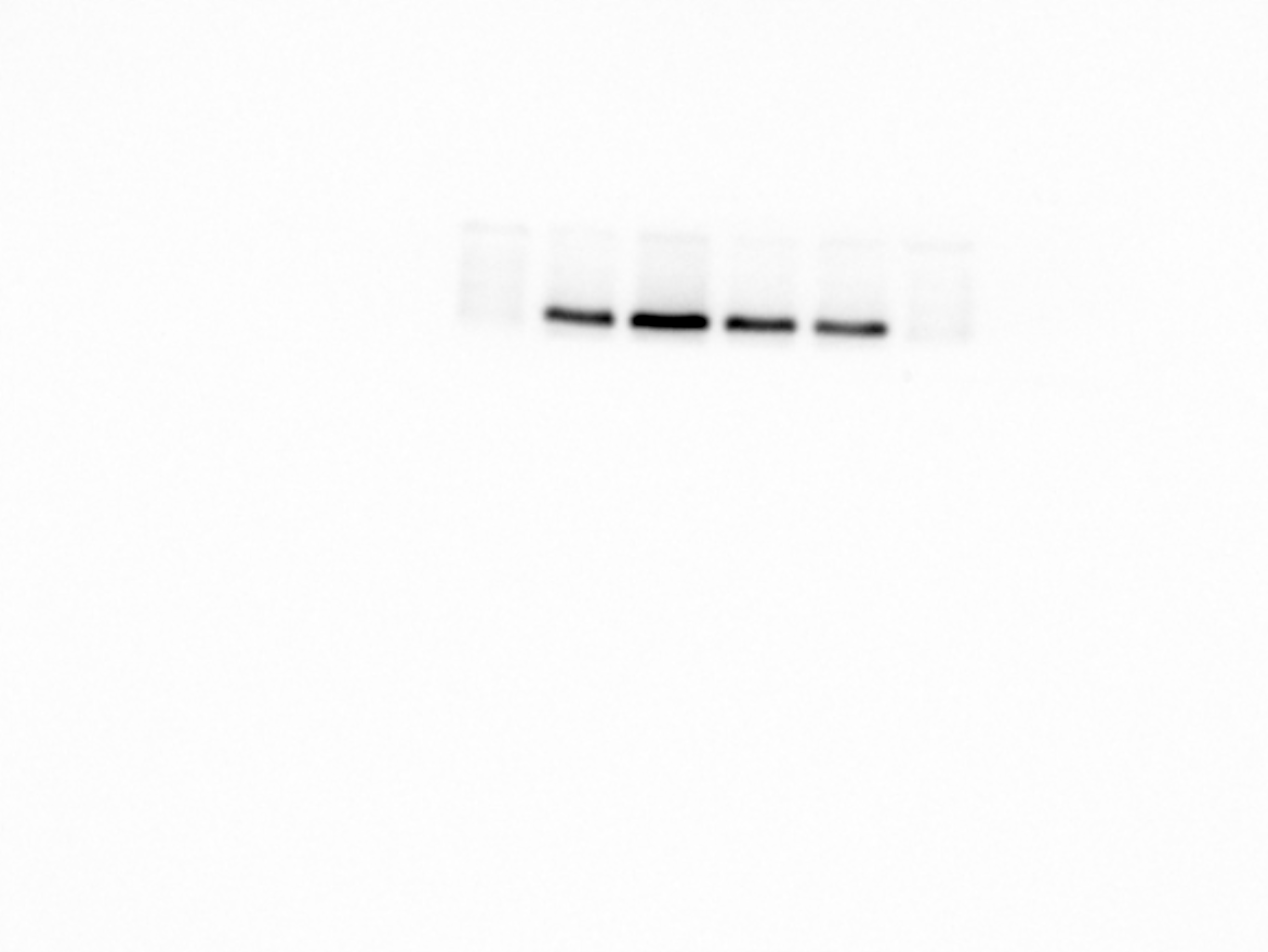 |
| 1 gapdh |  | Inside the red box is the image shown in Figure 4a |
| 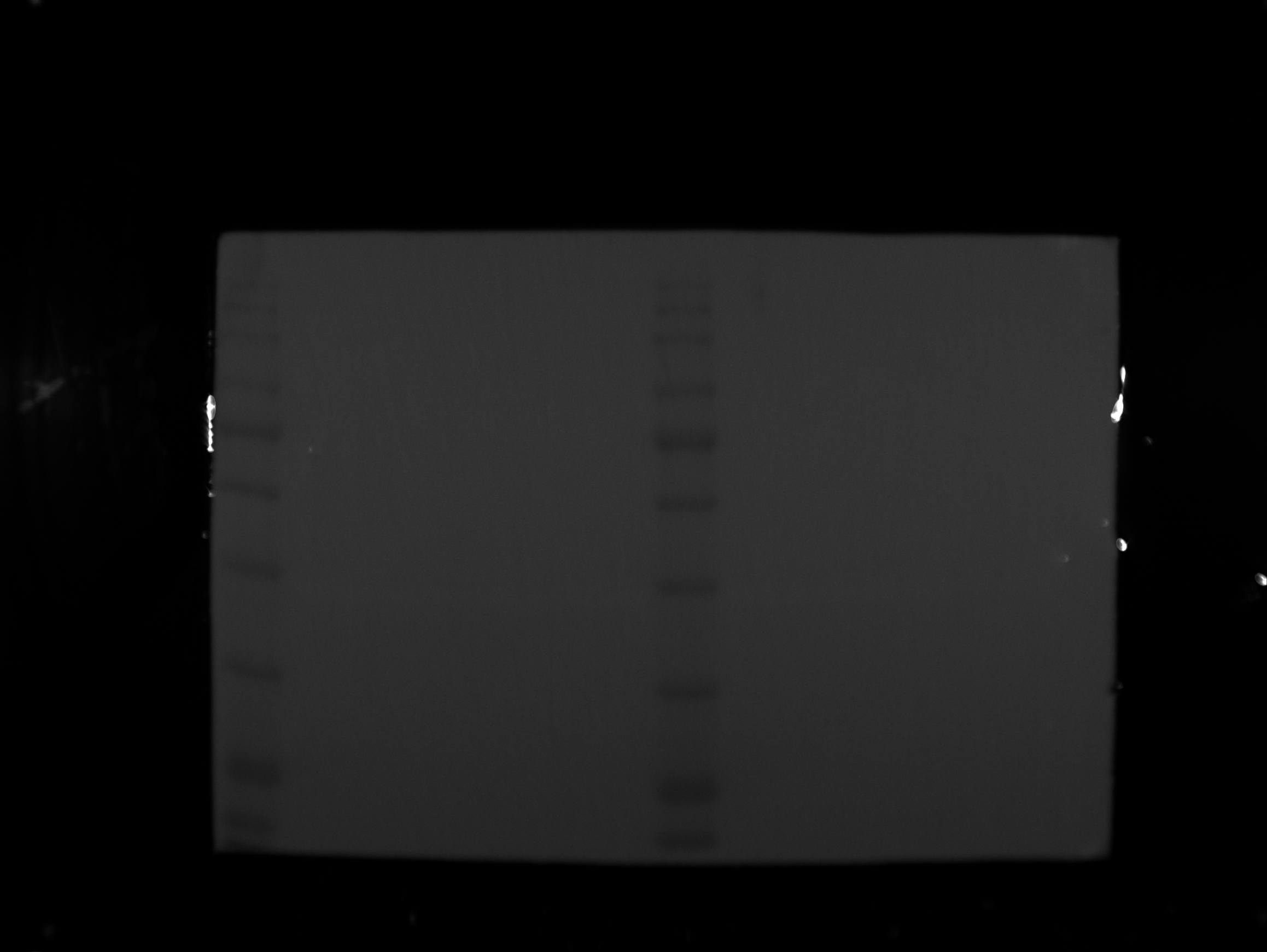 | 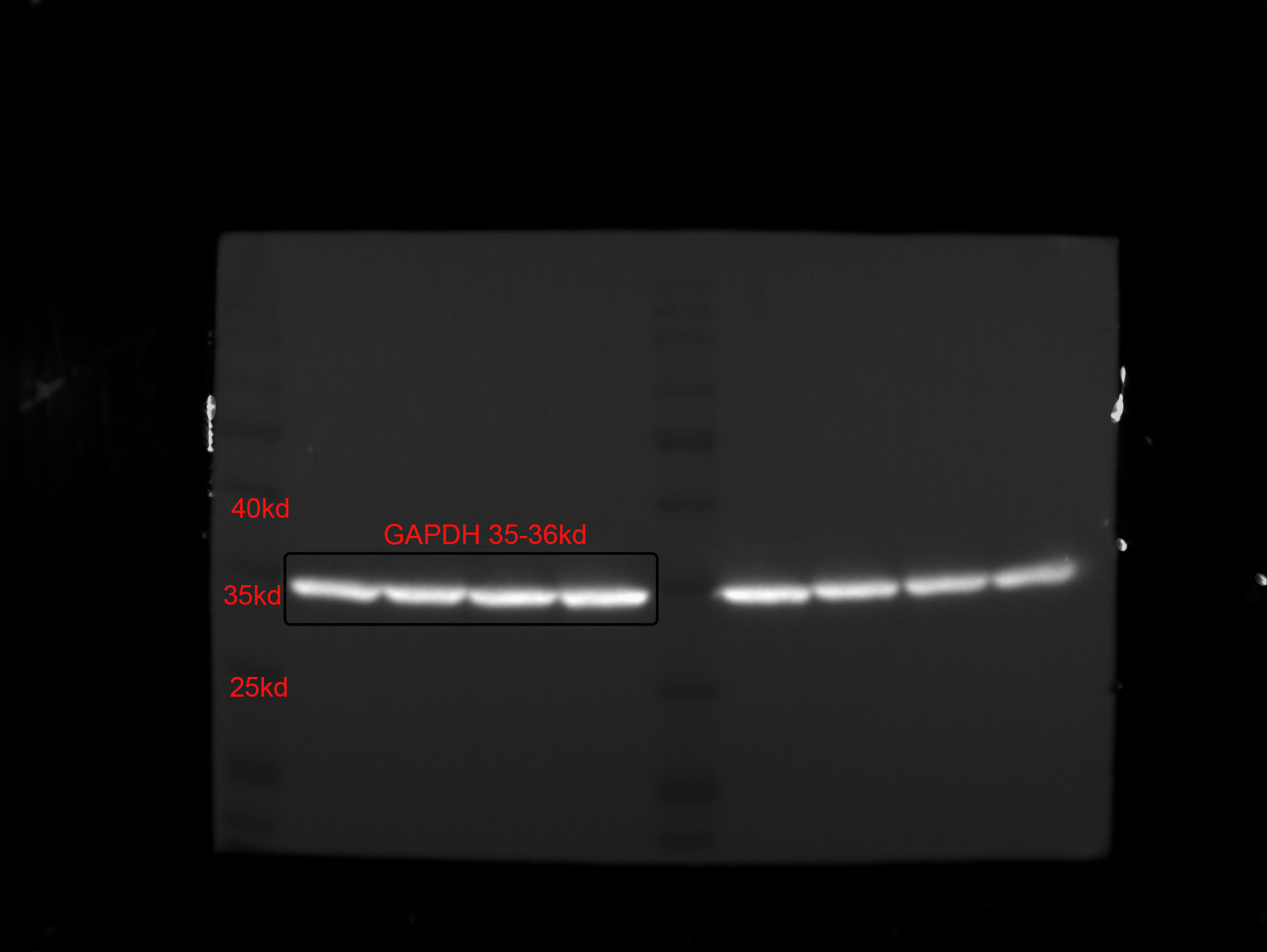 | 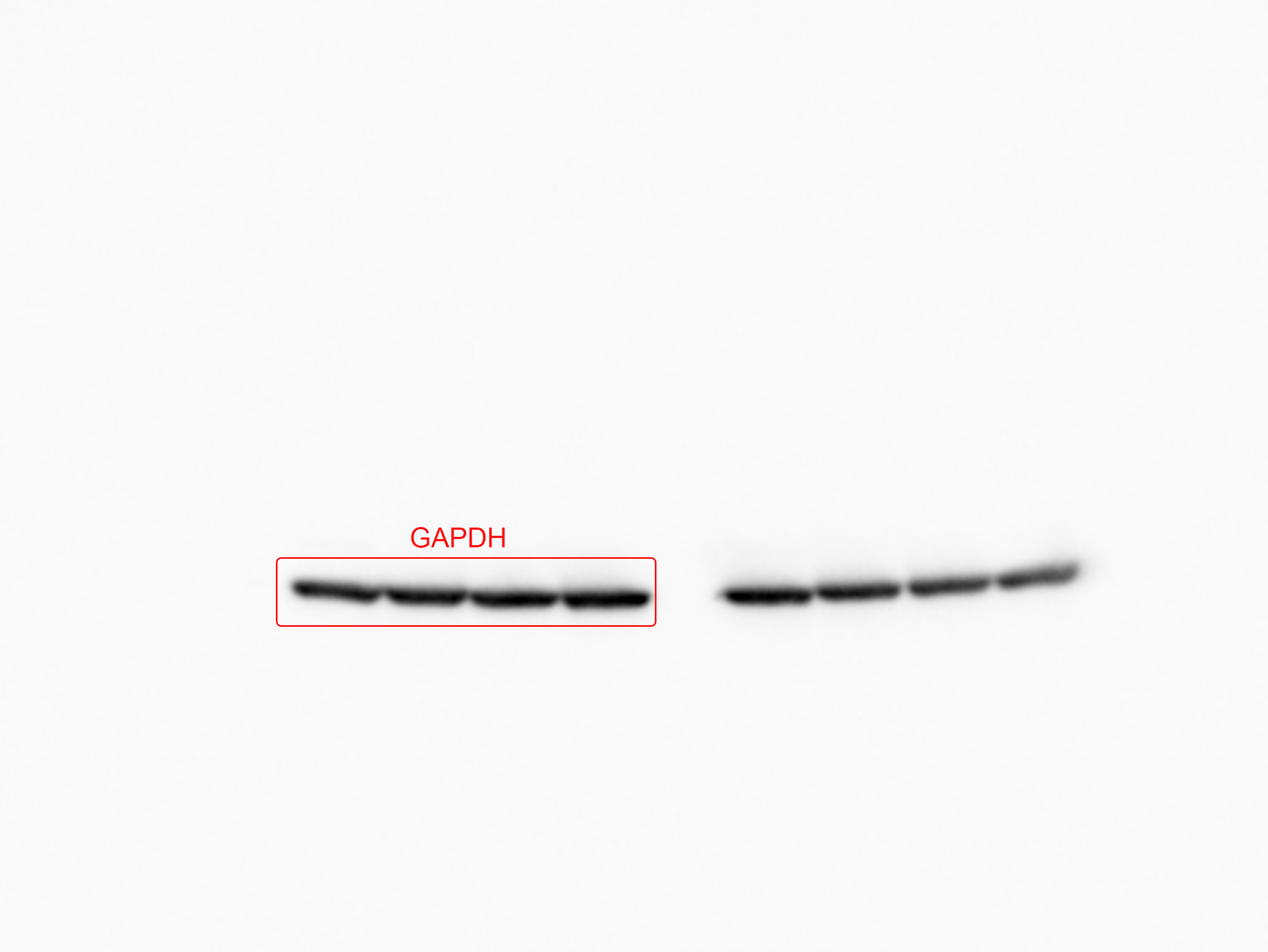 |

| 2 Bax |  |  |
| --- | --- | --- |
| 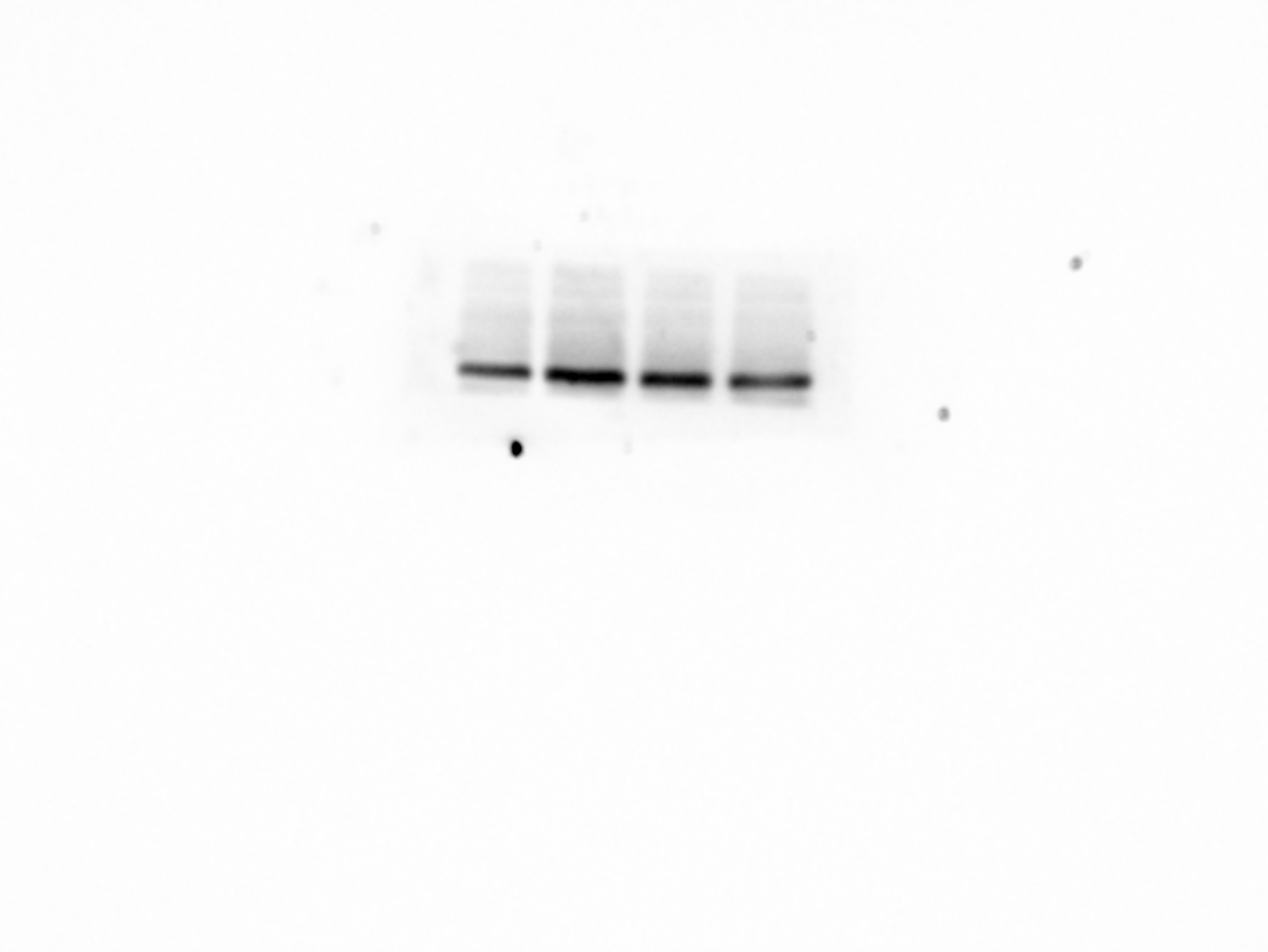 | 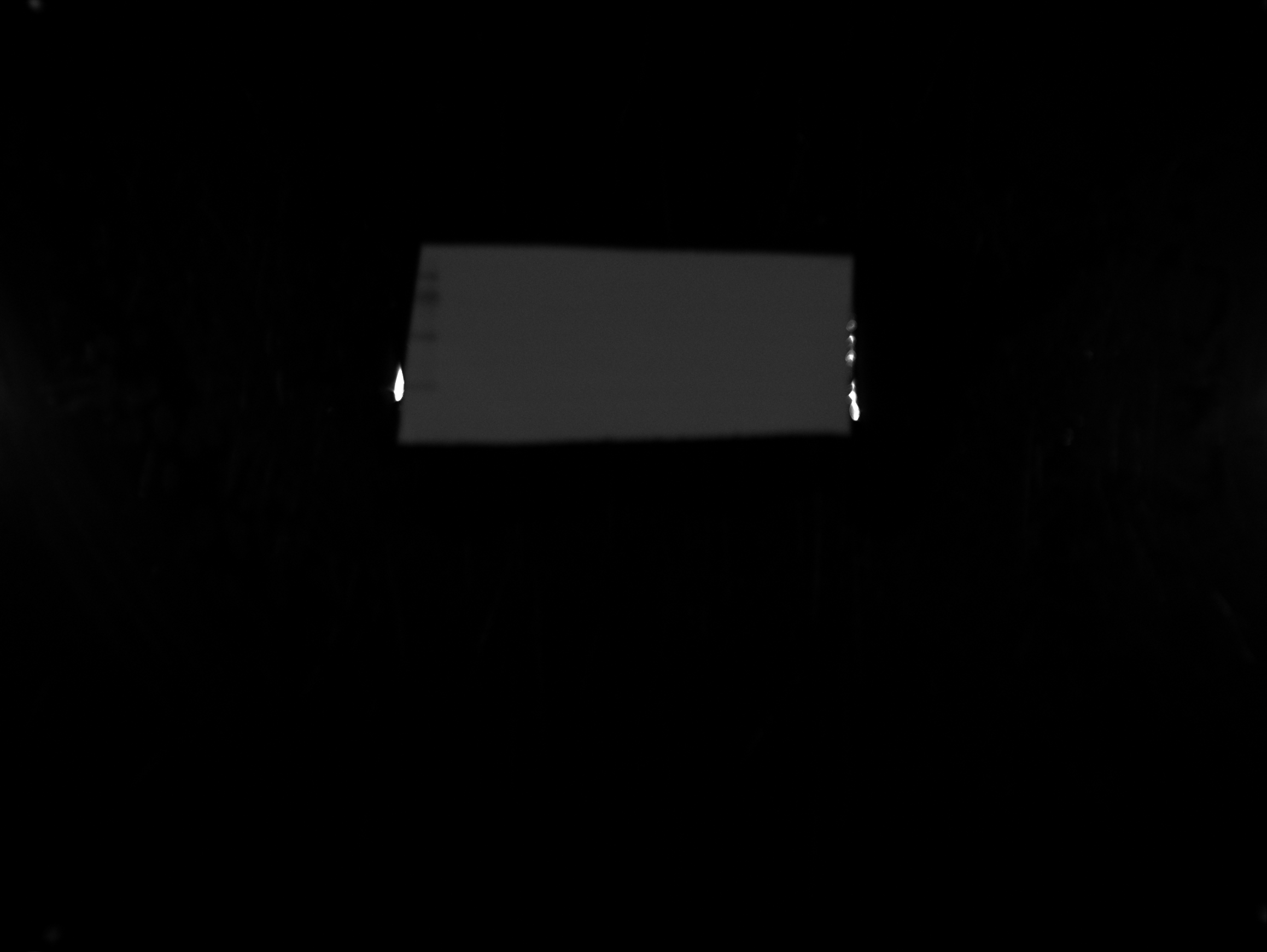 | 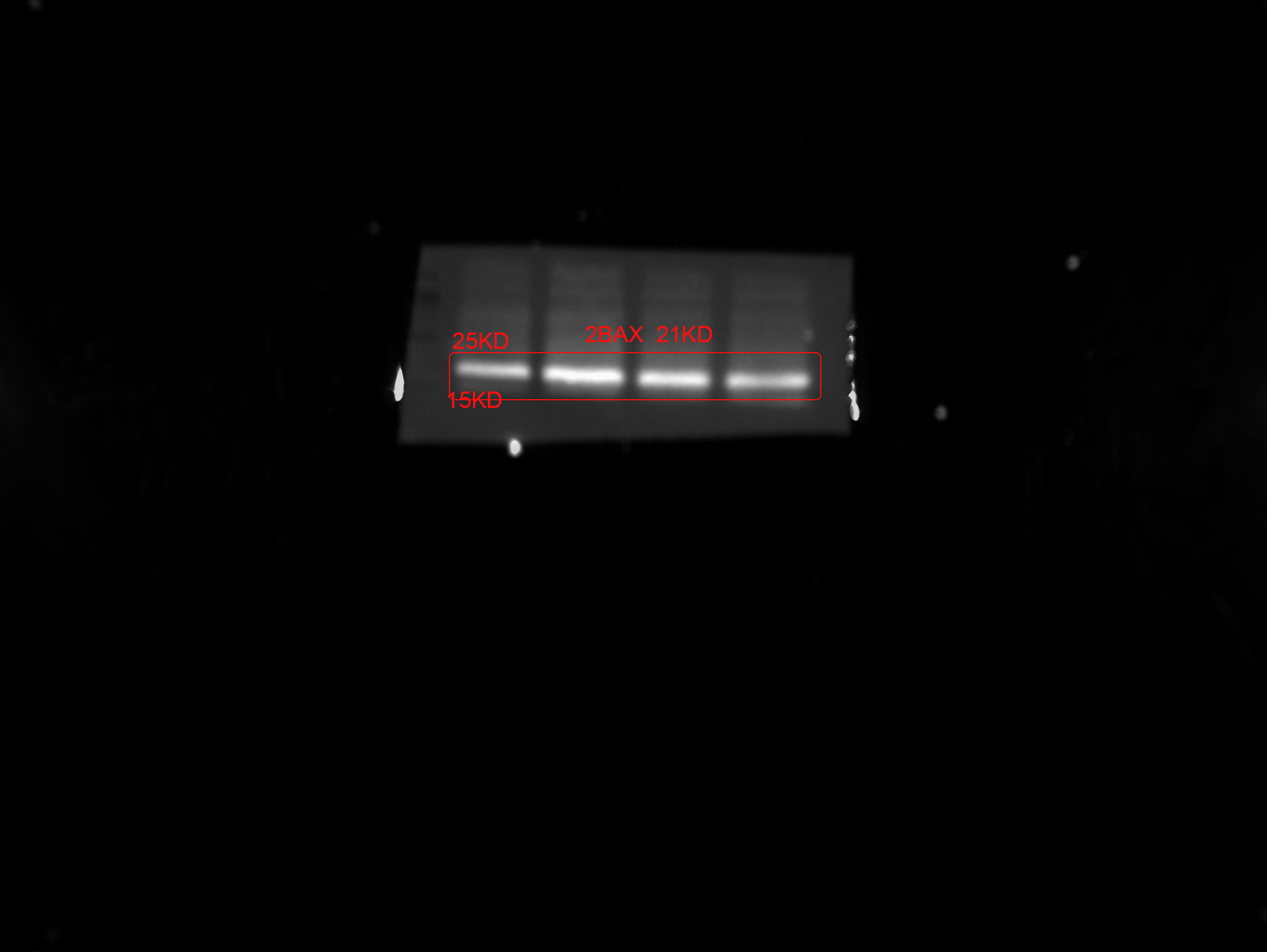 |
| 2 gapdh |  |  |
| 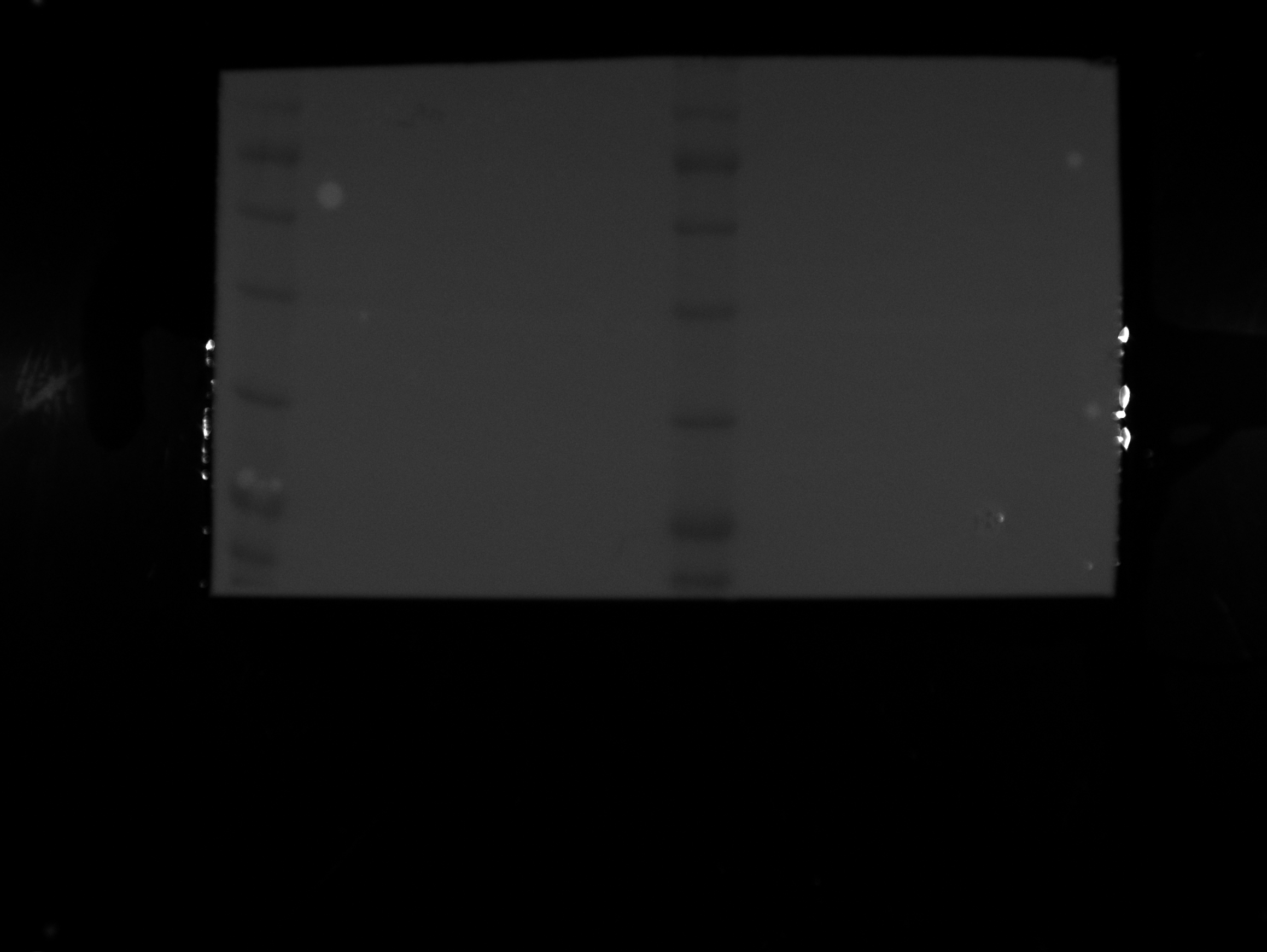 | 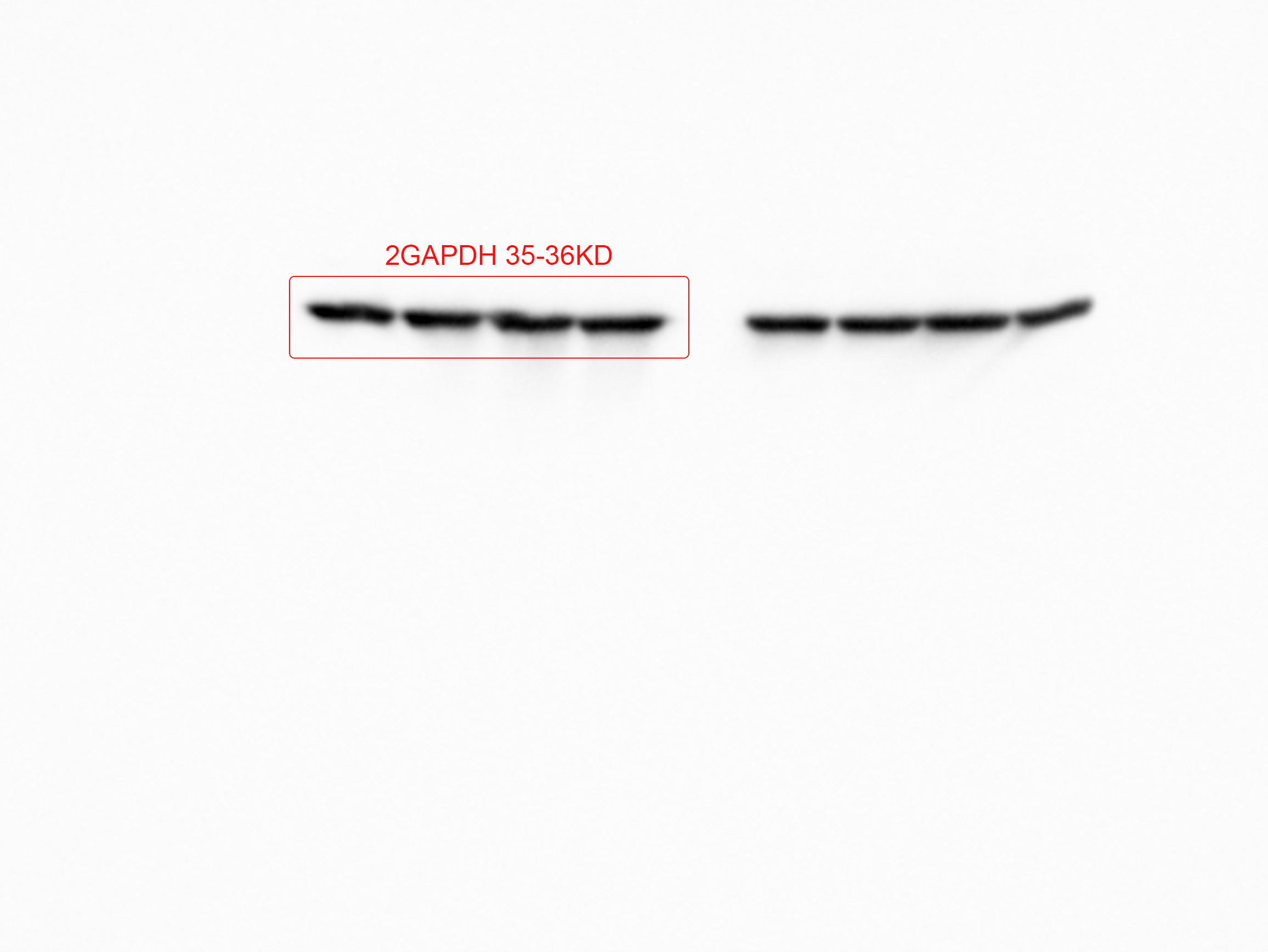 | 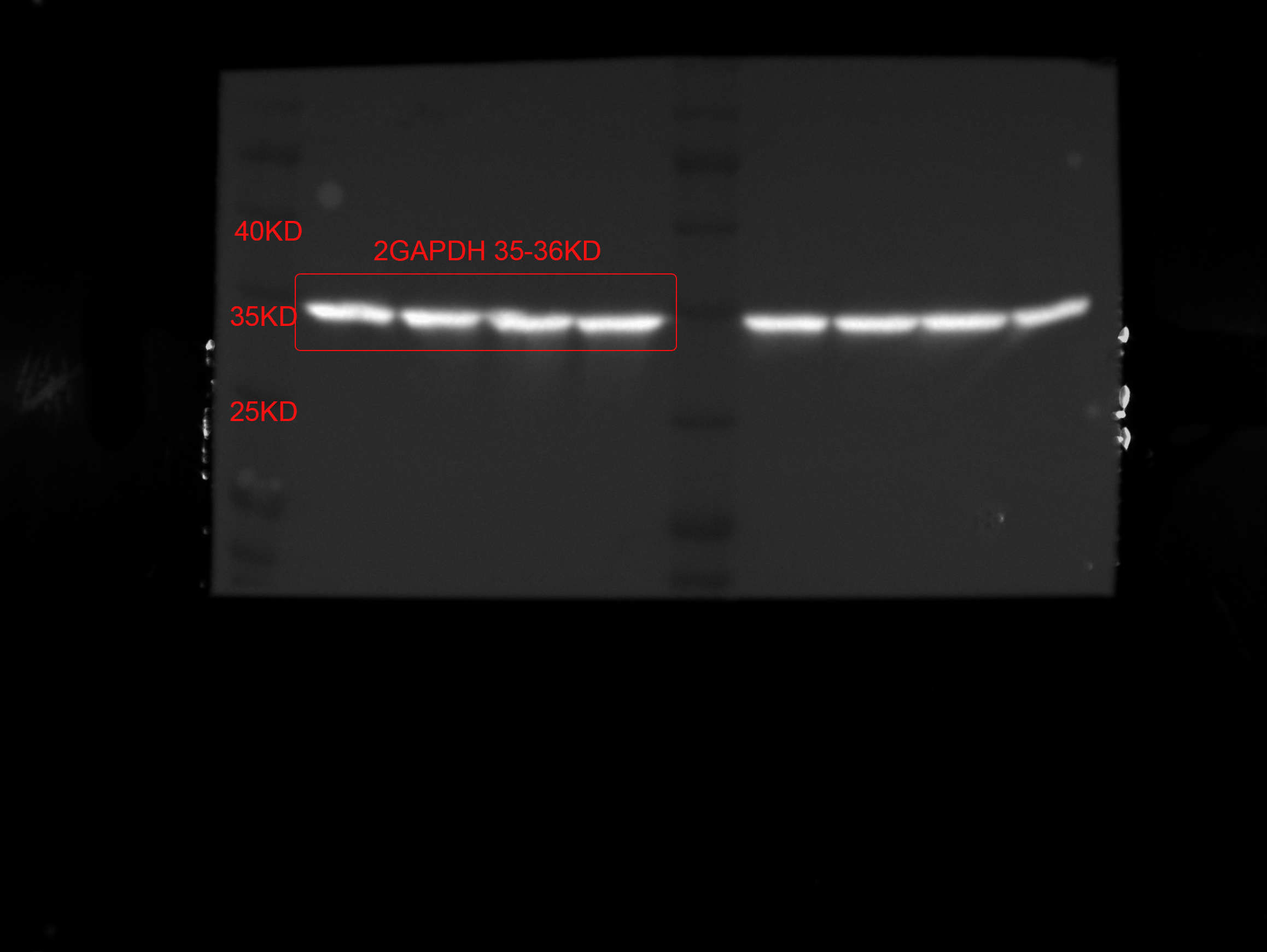 |

| 3 Bax | This Bax image is the one shown in Figure 4a |  |
| --- | --- | --- |
| 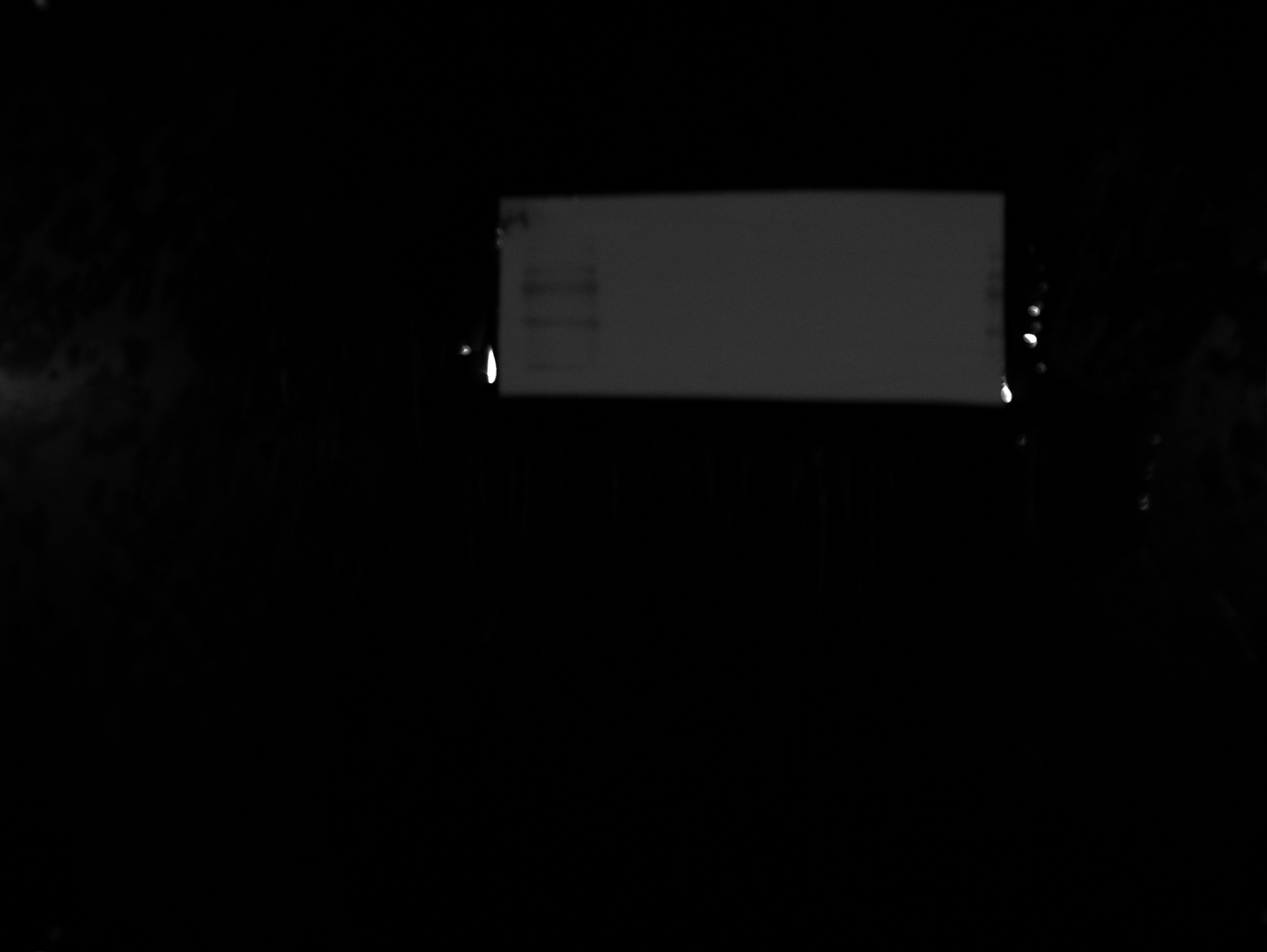 | 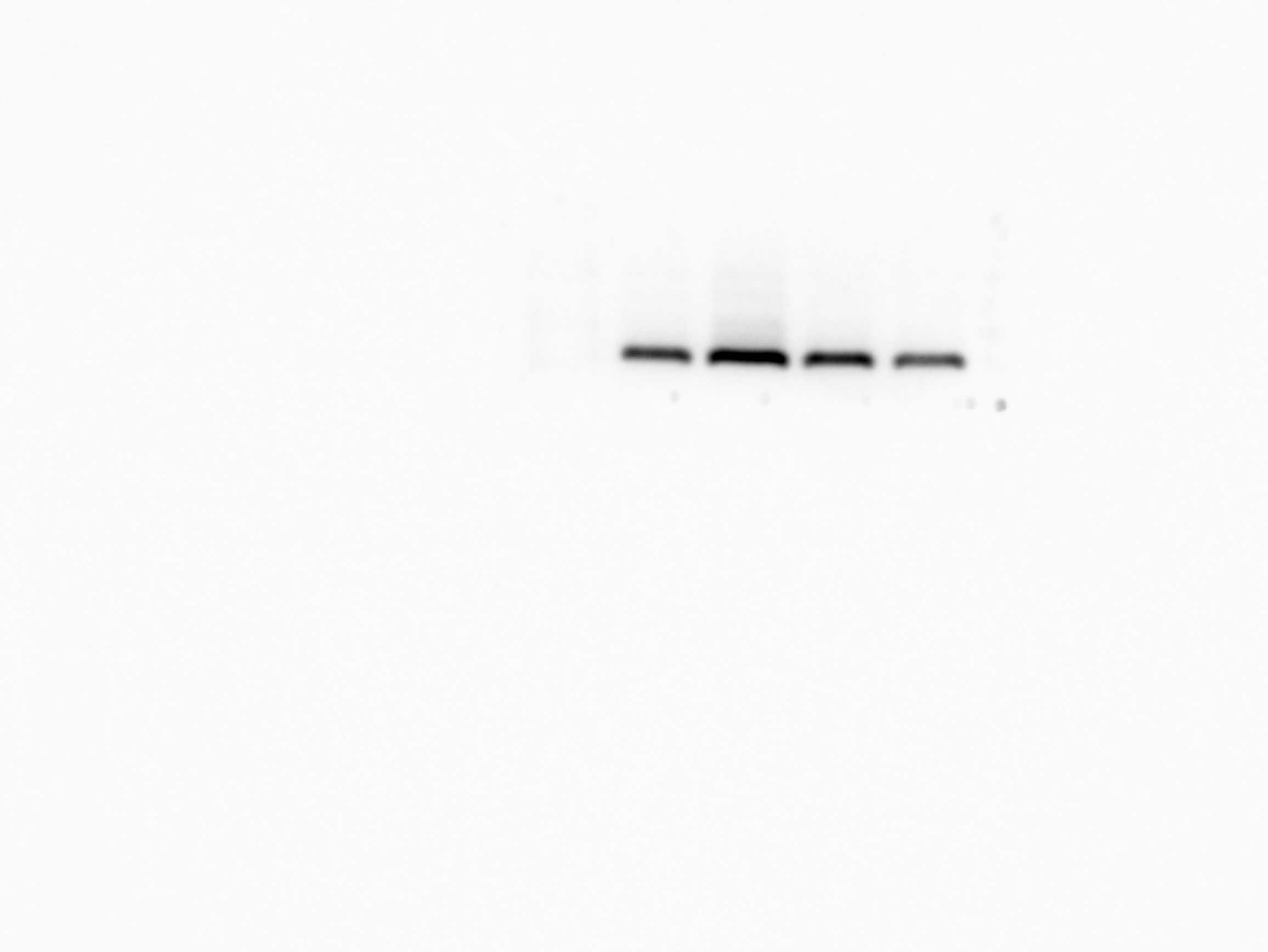 | 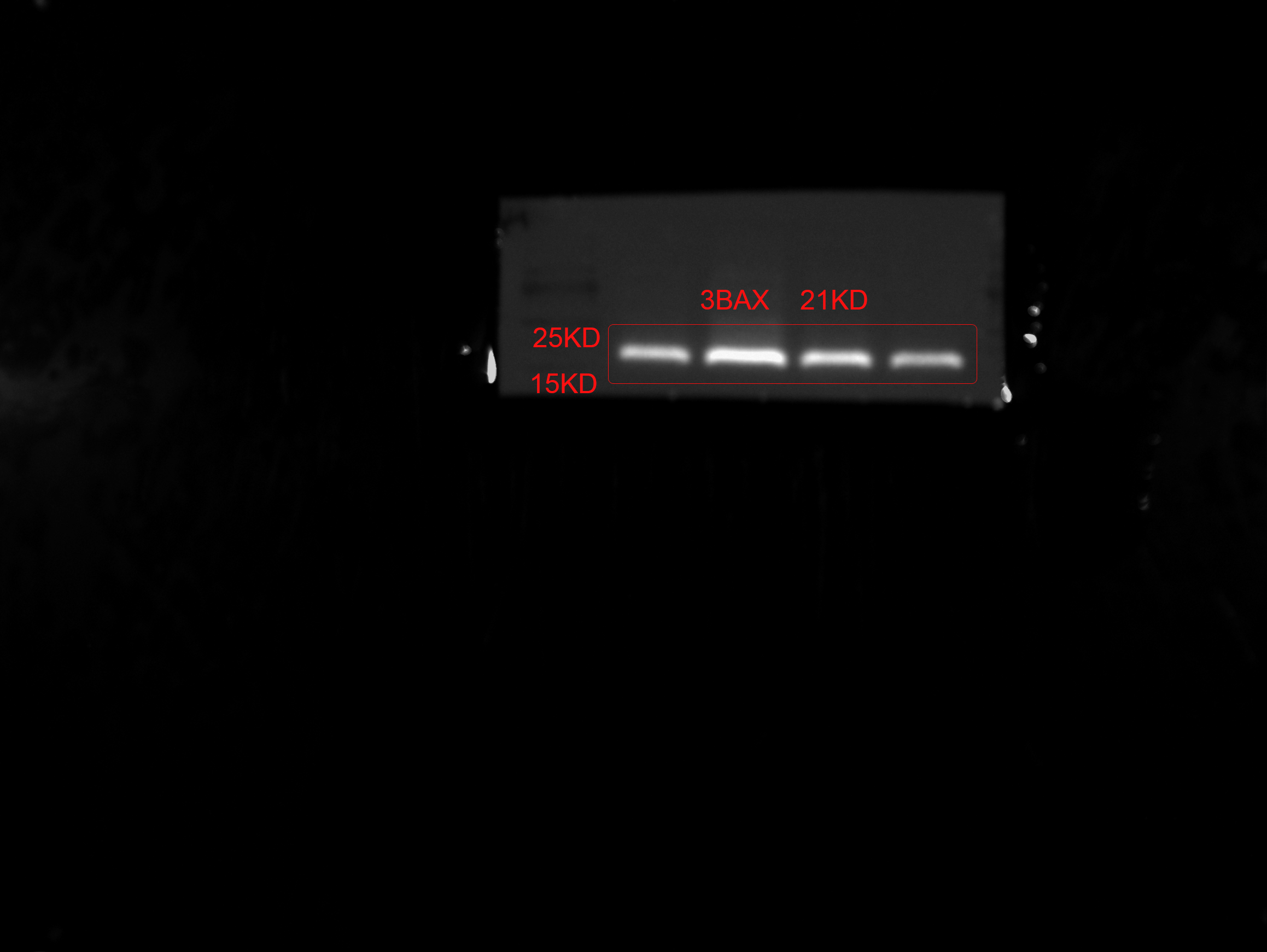 |
| 3gapdh |  |  |
| 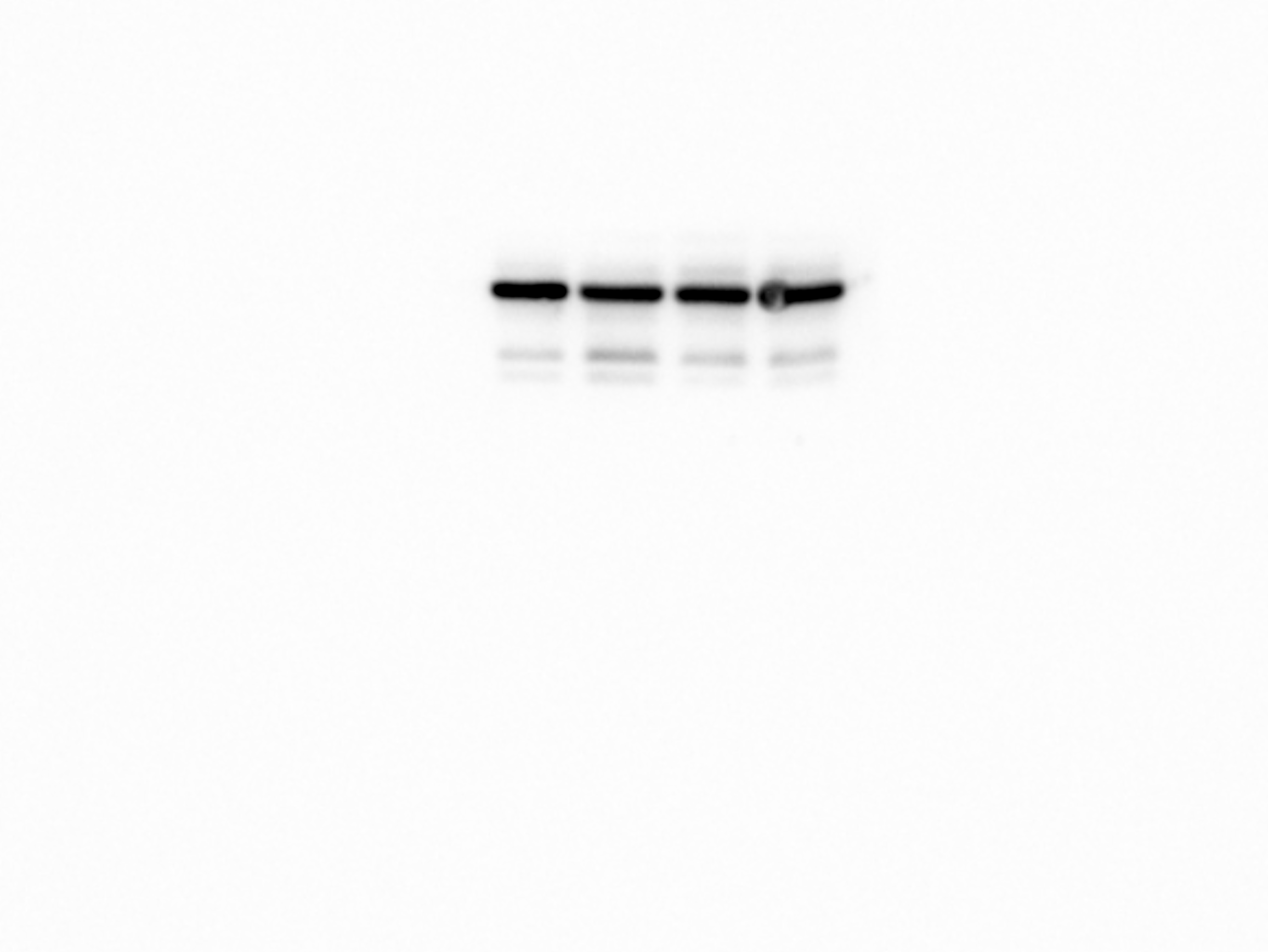 | 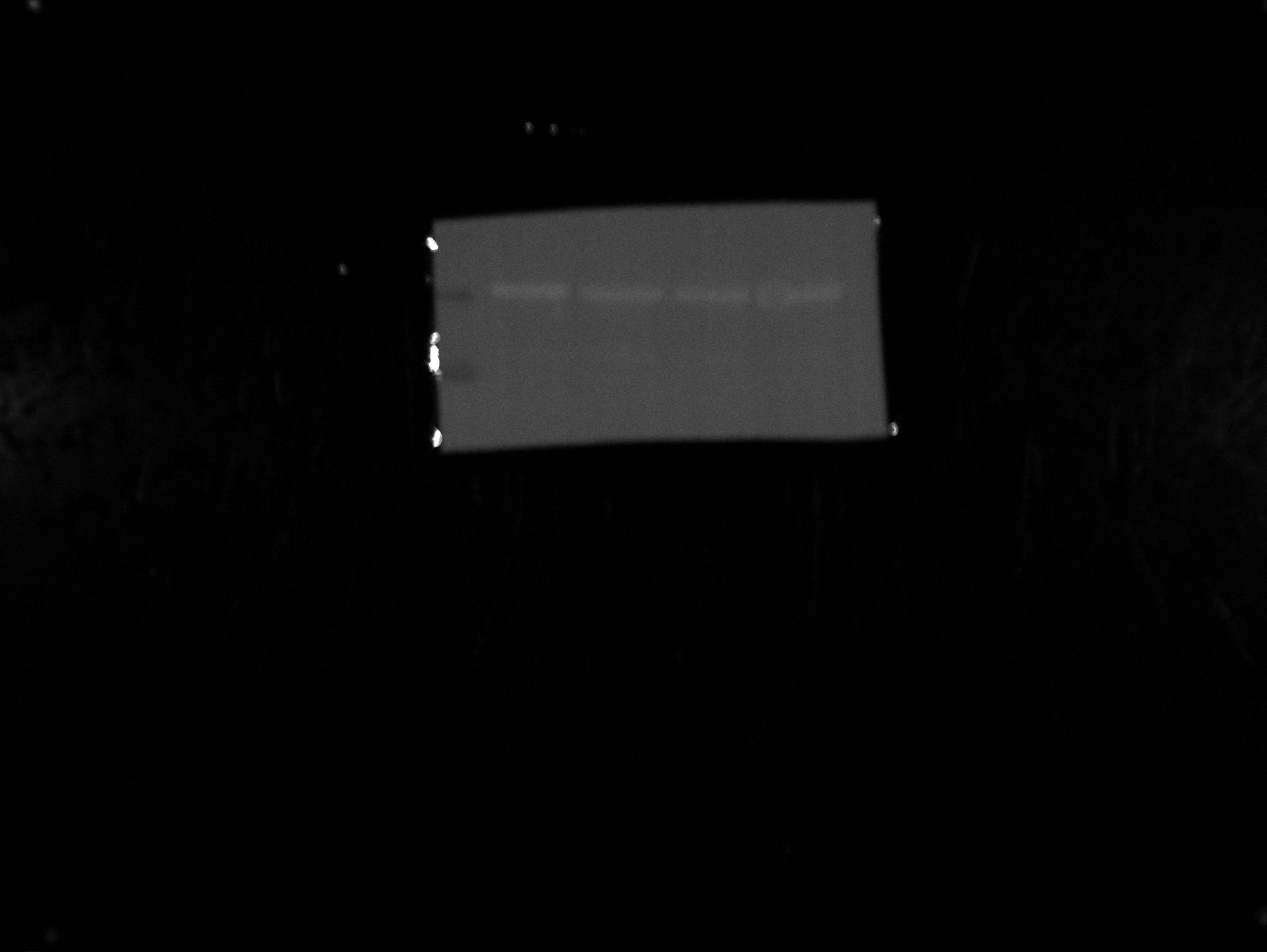 | 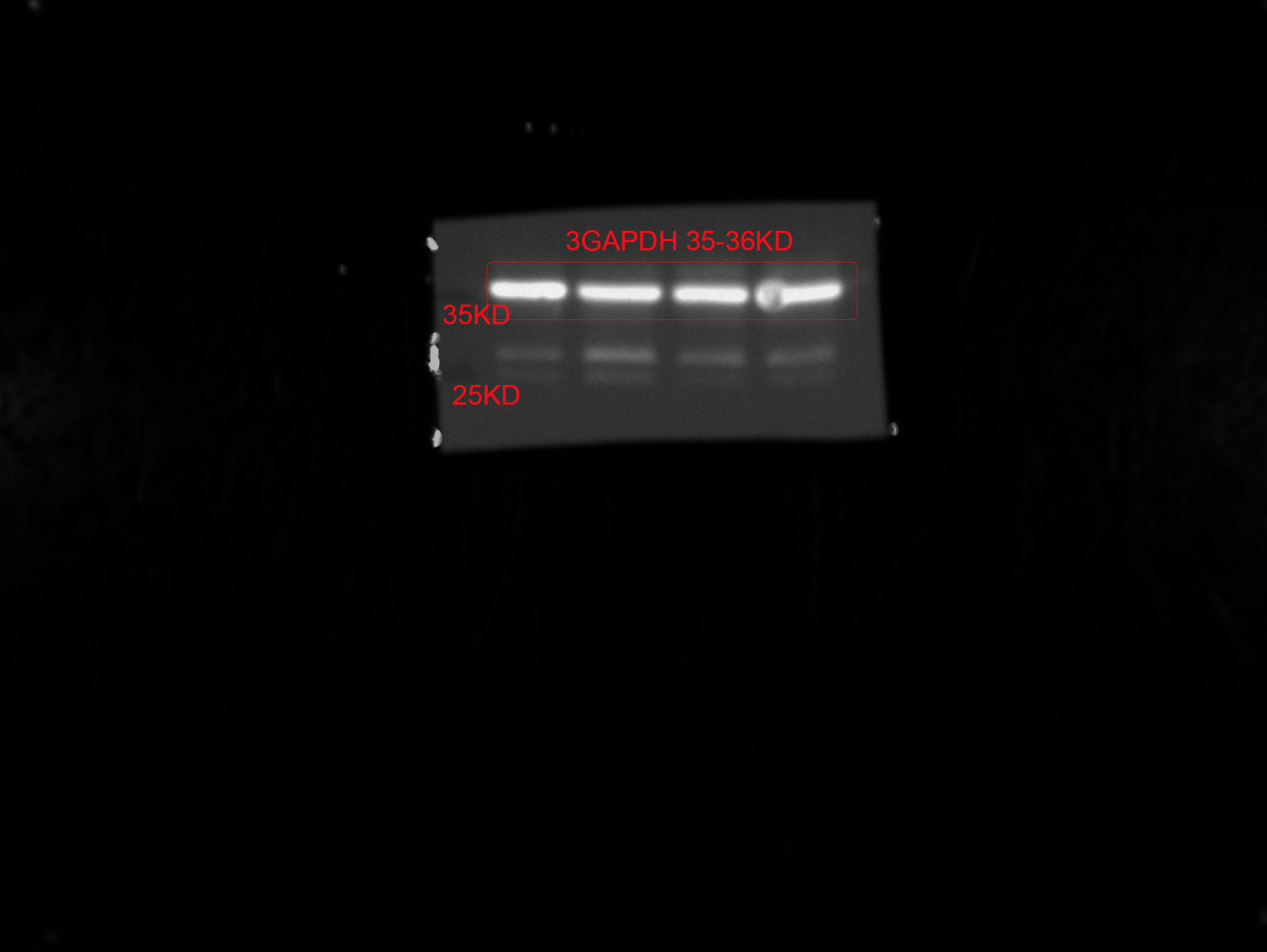 |
