# Supplementary material for: Amifostine attenuates bleomycin-induced pulmonary fibrosis in mice through inhibition of the PI3K/Akt/mTOR signaling pathway
Source: Sci Rep. 2023 Jun 28;13:10485. doi: 10.1038/s41598-023-34060-8 (PMC10307827; doi:10.1038/s41598-023-34060-8)
Supplement: Supplementary file 2 — Supplementary Information 2. [file 41598_2023_34060_MOESM2_ESM.docx]

| 1 Bcl-2 |  |  |
| --- | --- | --- |
| 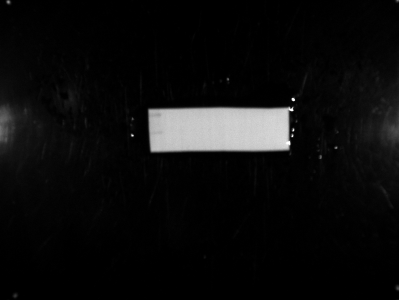 | 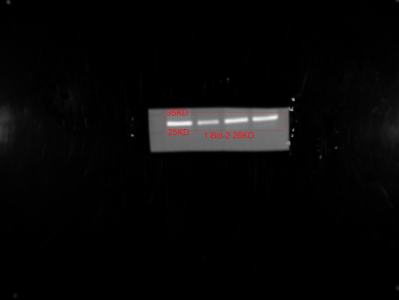 | 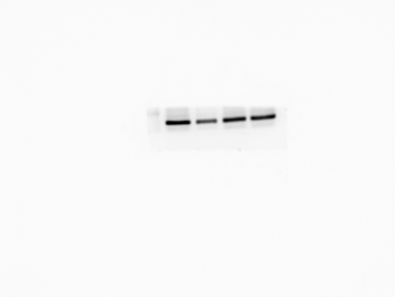 |
| 1gapdh |  |  |
| 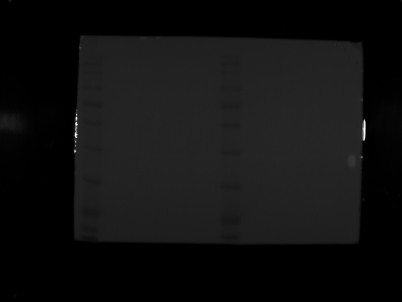 | 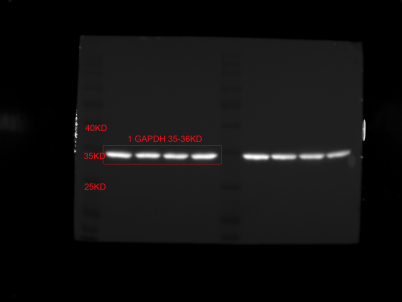 | 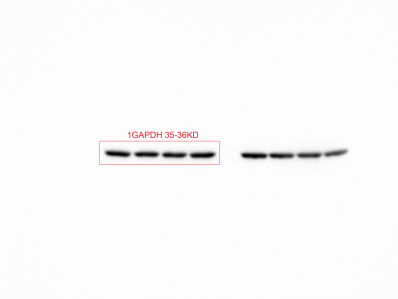 |

| 2 Bcl-2 This Bcl-2 image is the one shown in Figure 4a |  |  |
| --- | --- | --- |
| 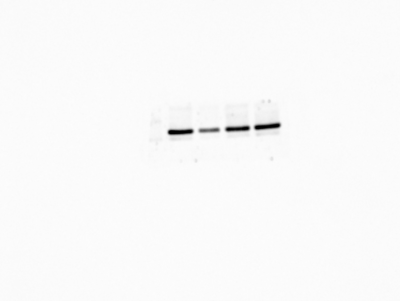 | 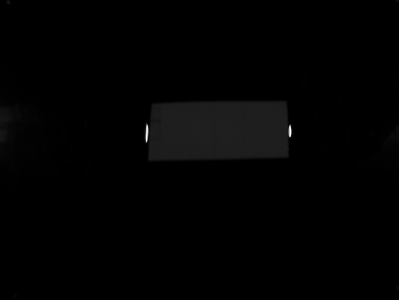 | 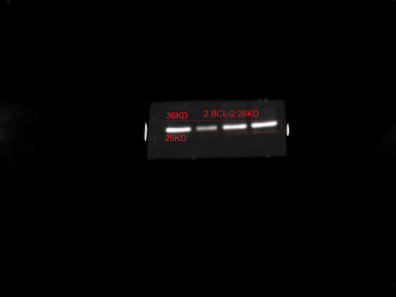 |
| 2gapdh |  |  |
| 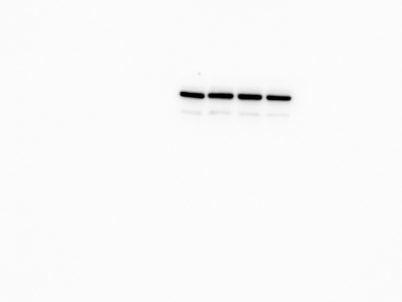 | 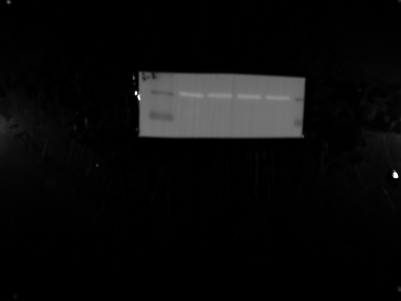 | 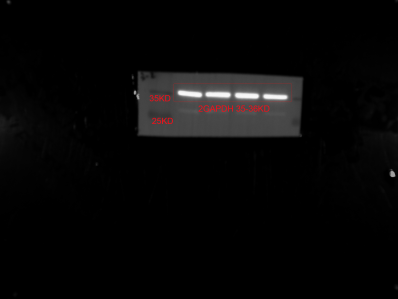 |

| 3 Bcl-2 |  |  |
| --- | --- | --- |
| 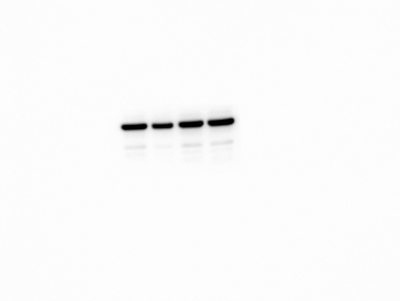 | 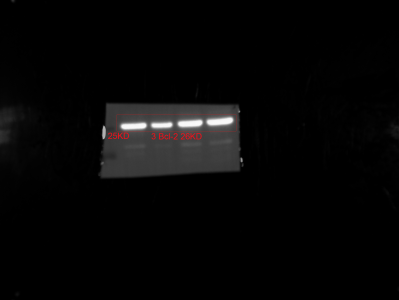 | 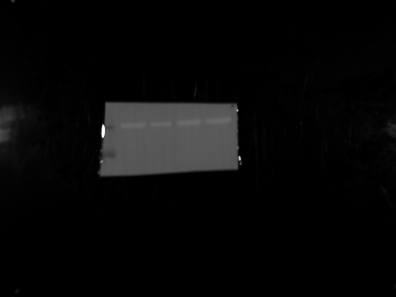 |
| 3gapdh The one on the right is the target band |  |  |
| 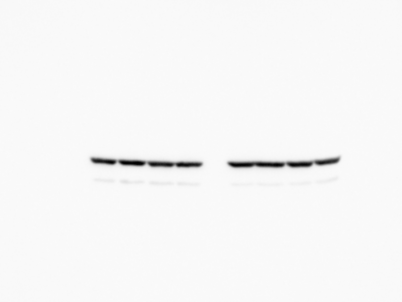 | 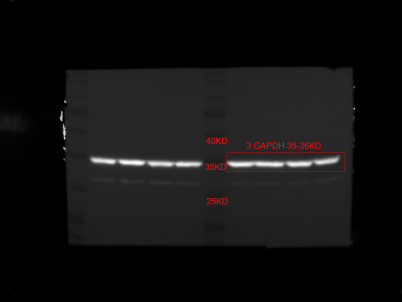 | 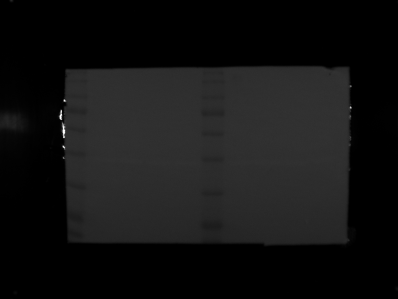 |
