# Supplementary material for: Amifostine attenuates bleomycin-induced pulmonary fibrosis in mice through inhibition of the PI3K/Akt/mTOR signaling pathway
Source: Sci Rep. 2023 Jun 28;13:10485. doi: 10.1038/s41598-023-34060-8 (PMC10307827; doi:10.1038/s41598-023-34060-8)
Supplement: Supplementary file 9 — Supplementary Information 9. [file 41598_2023_34060_MOESM9_ESM.docx]

| 1p-AKT |  | The image in the red frame on the left is the image shown by p-AKT in Figure 6a |
| --- | --- | --- |
| 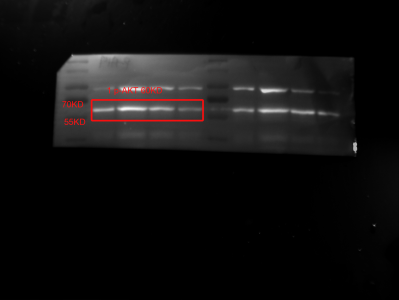 | 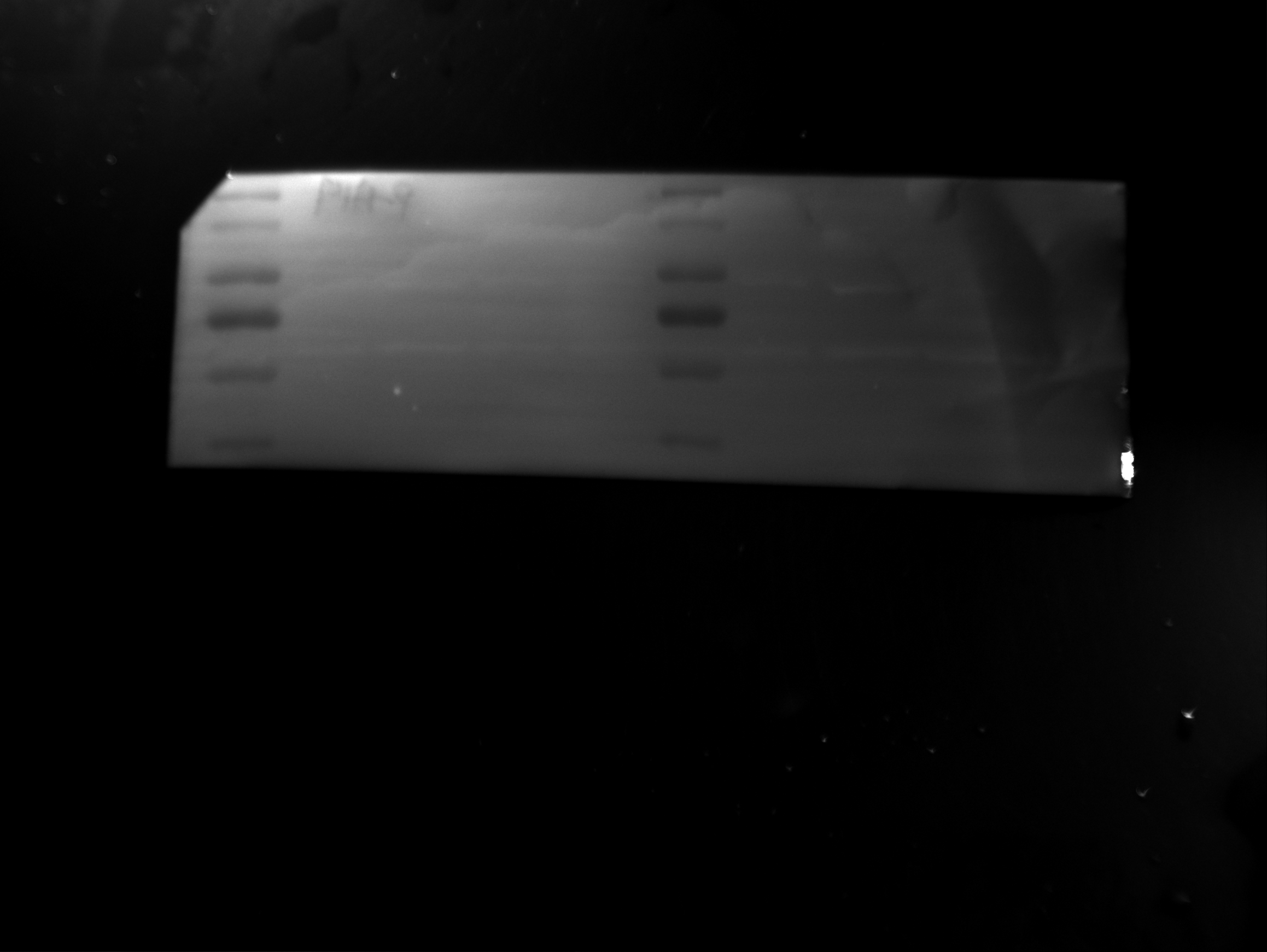 | 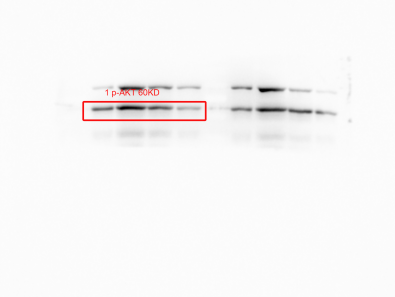 |
| 1gapdh |  |  |
| 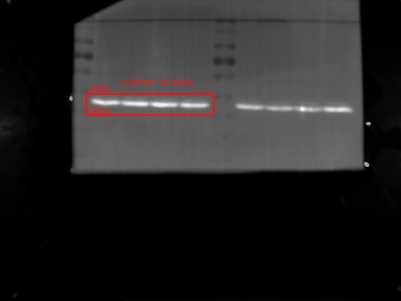 | 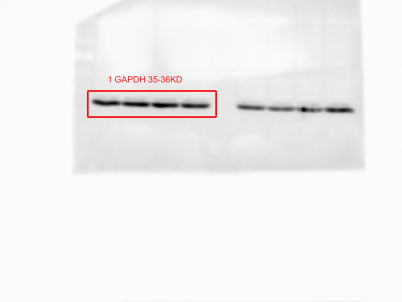 | 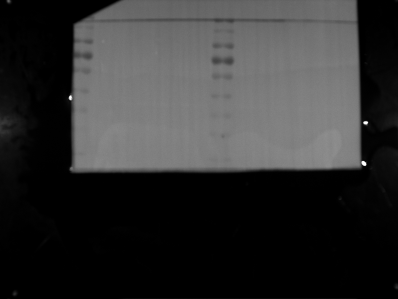 |

| 2p-AKT |  |  |
| --- | --- | --- |
| 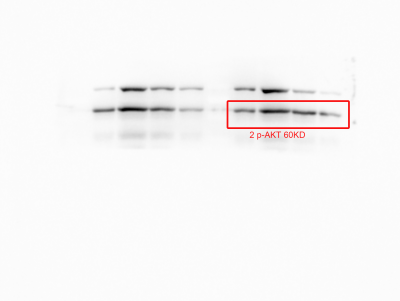 | 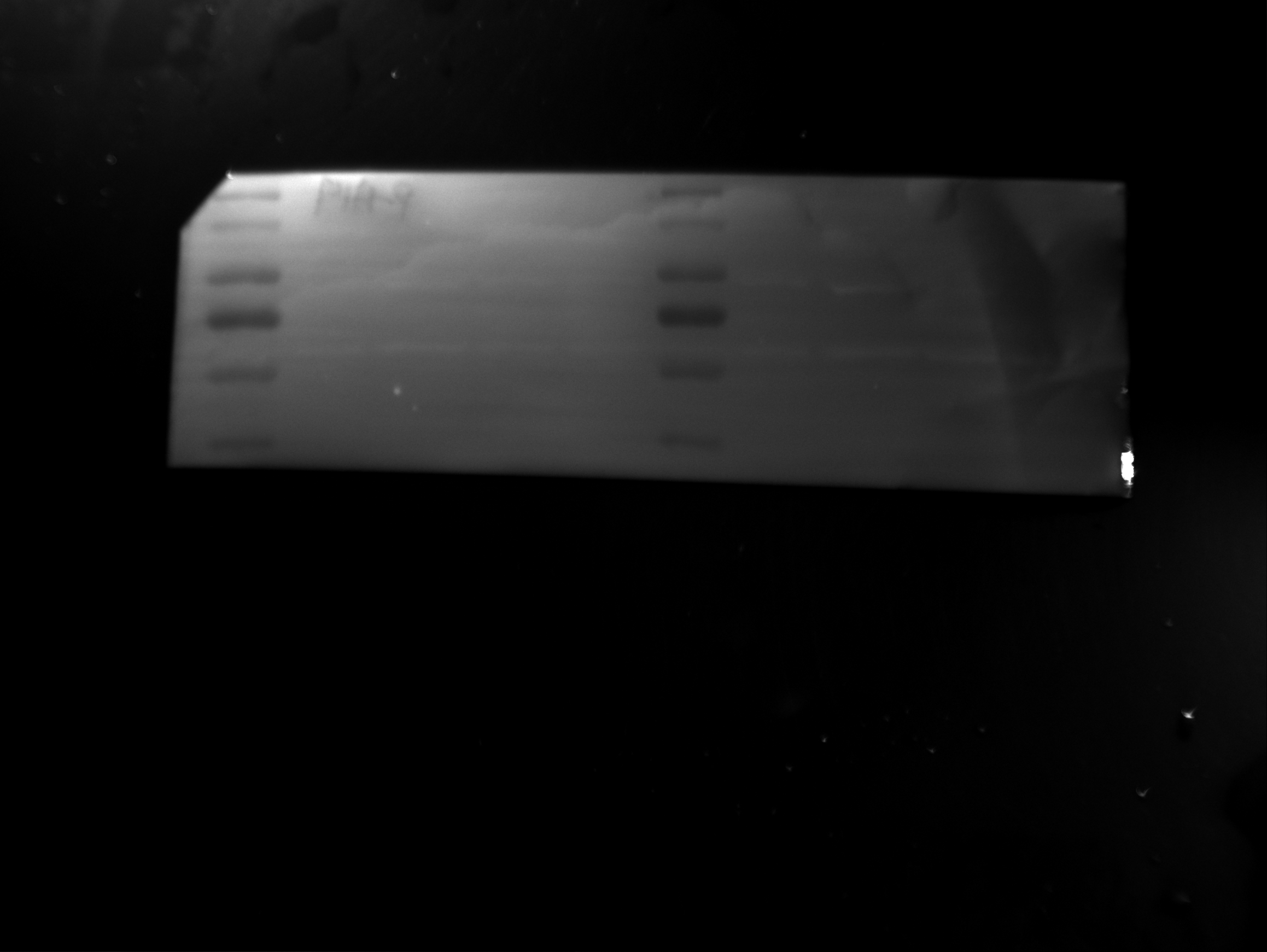 | 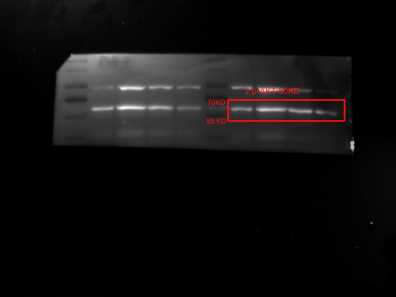 |
| 2gapdh |  | The image in the red frame on the right is the image shown by GAPDH in Figure 6a |
| 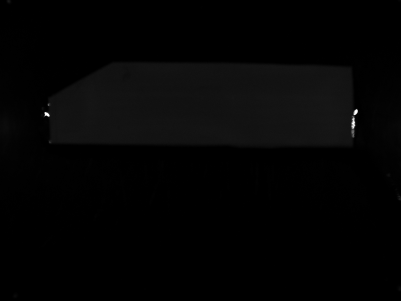 | 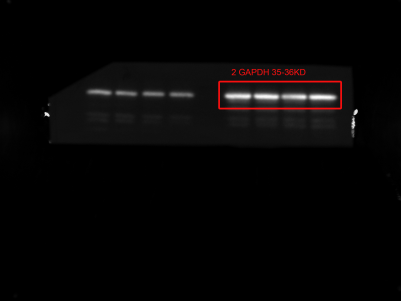 | 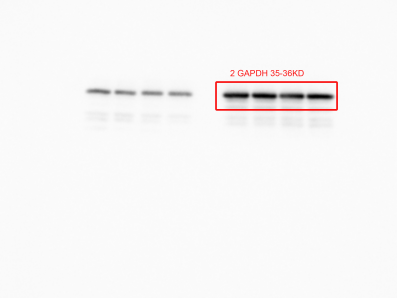 |

| 3p-AKT |  |  |
| --- | --- | --- |
| 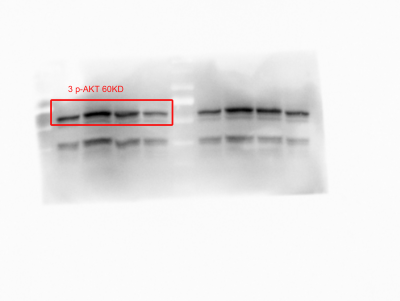 | 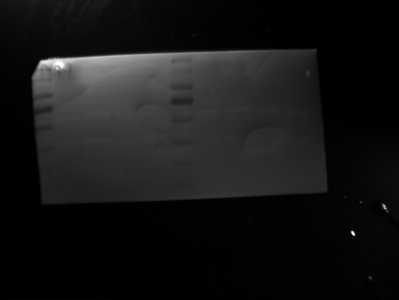 | 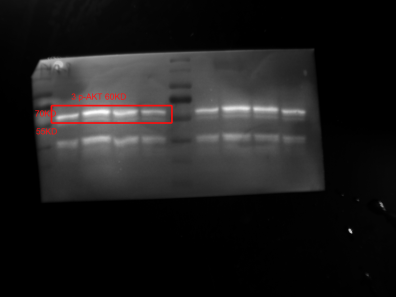 |
| 3gapdh |  |  |
| 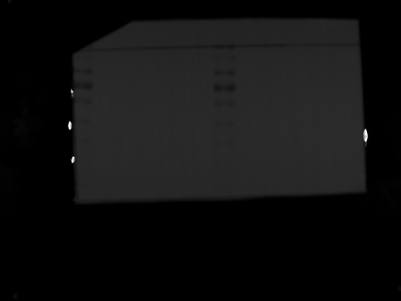 | 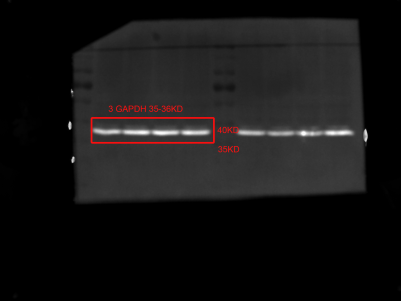 | 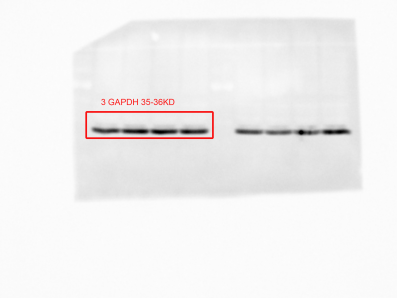 |
