# Supplementary figures and images for: Amifostine attenuates bleomycin-induced pulmonary fibrosis in mice through inhibition of the PI3K/Akt/mTOR signaling pathway
Source: Sci Rep. 2023 Jun 28;13:10485. doi: 10.1038/s41598-023-34060-8 (PMC10307827; doi:10.1038/s41598-023-34060-8)

| 1p-mTOR |  |  |
| --- | --- | --- |
| 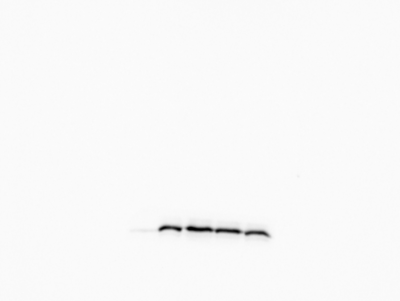 | 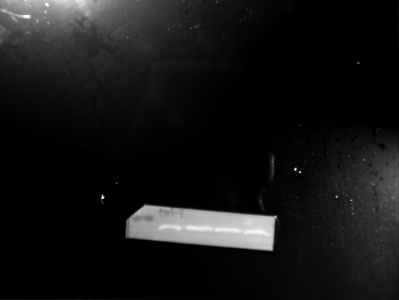 | 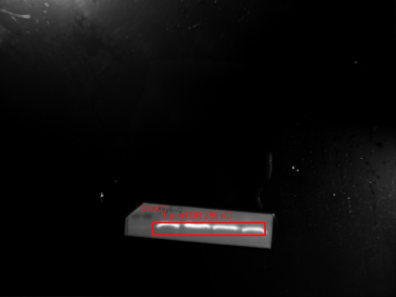 |
| 1gapdh |  |  |
| 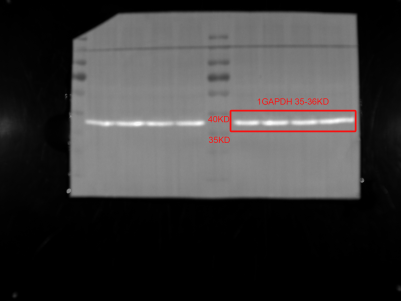 | 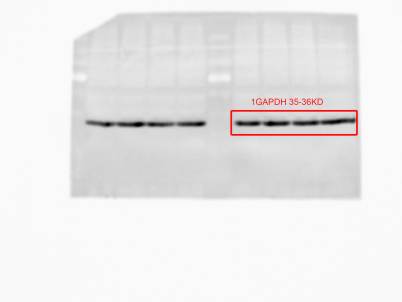 | 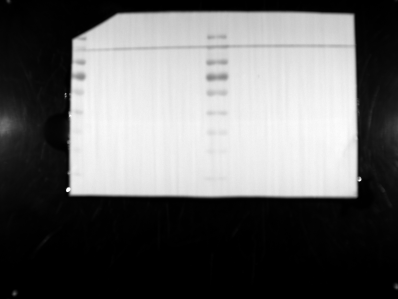 |

| 2p-mTOR |  | This image is the image shown by p-mTOR in Figure 6a |
| --- | --- | --- |
| 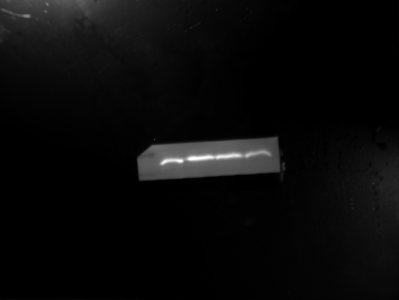 | 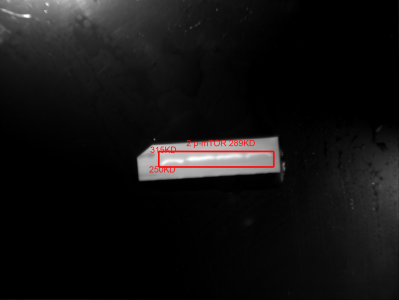 | 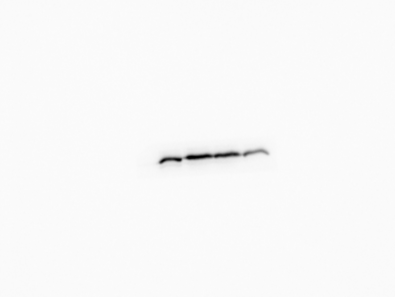 |
| 2gapdh |  |  |
| 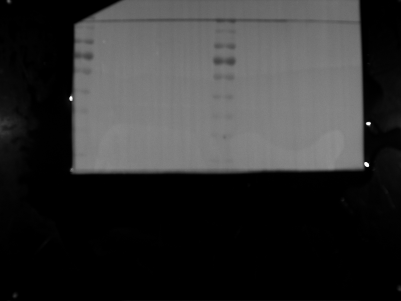 | 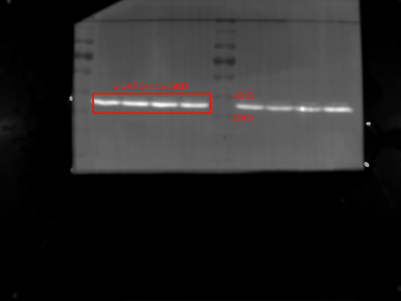 | 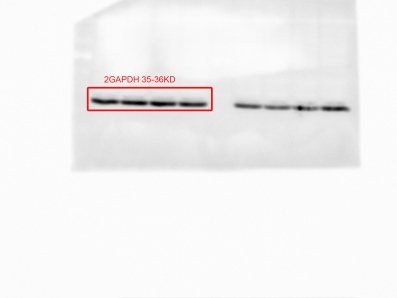 |

| 3p-mTOR |  |  |
| --- | --- | --- |
| 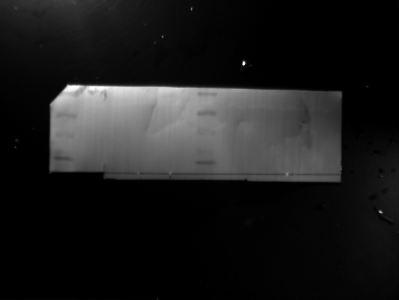 | 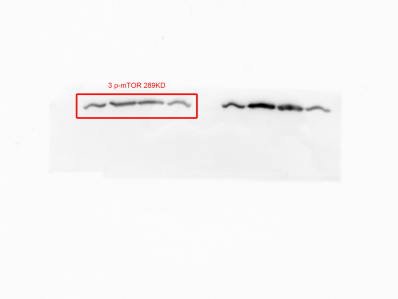 | 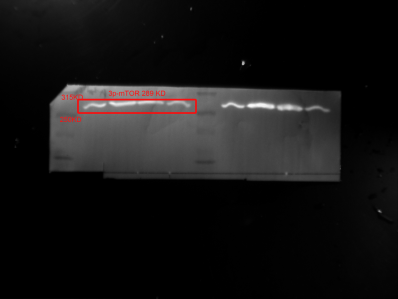 |
| 3gapdh |  |  |
| 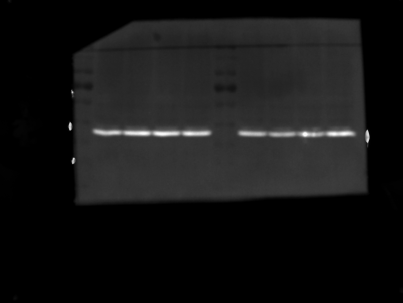 | 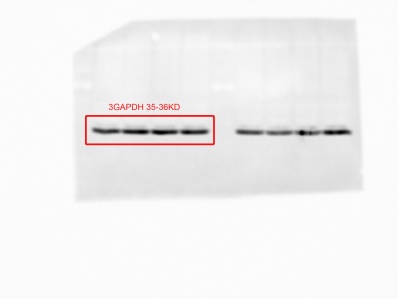 | 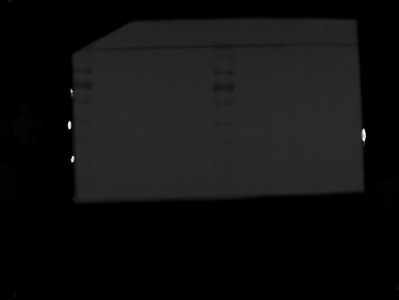 |
|  |  |  |

Supplement: Supplementary file 10 — Supplementary Information 10. [file 41598_2023_34060_MOESM10_ESM.docx]

| 1p-PI3K |  | This image is the image shown by p-PI3K in Figure 6a |
| --- | --- | --- |
| 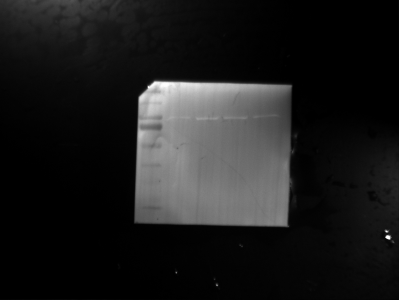 | 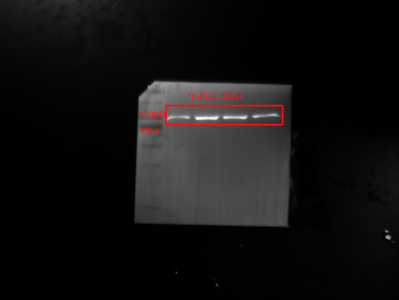 | 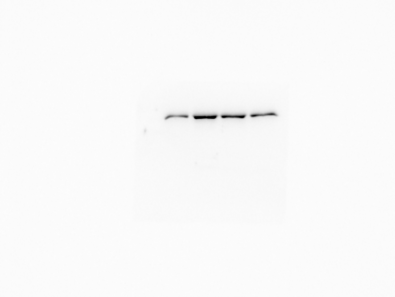 |
| 1gapdh |  |  |
| 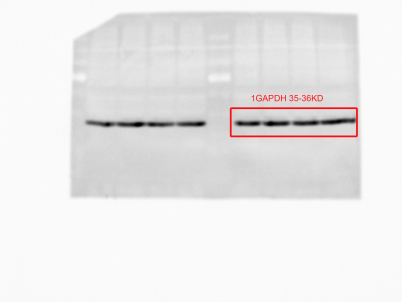 | 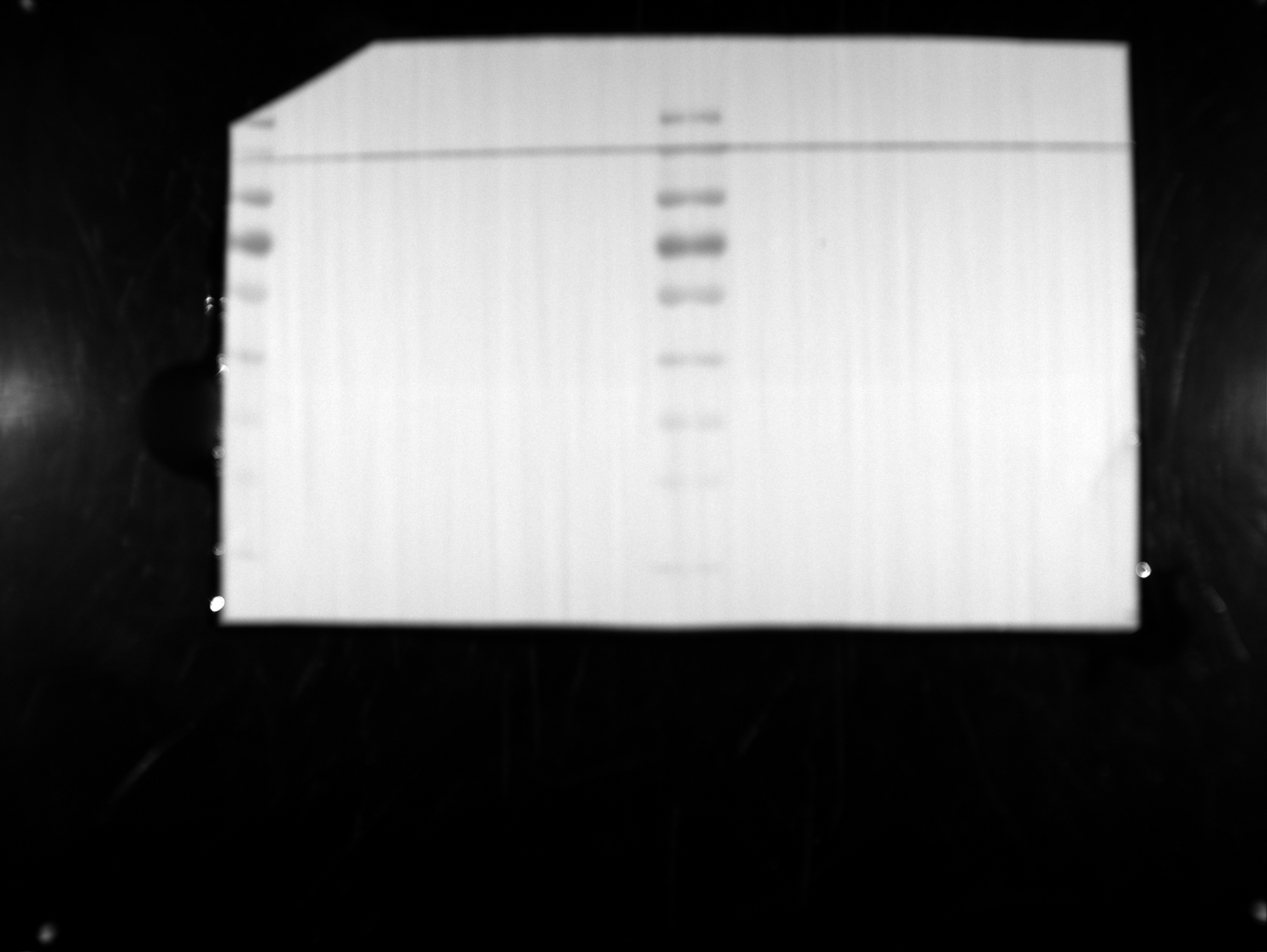 | 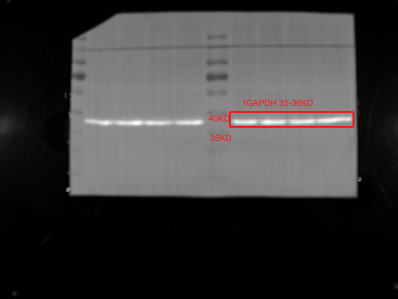 |

| 2p-PI3K |  |  |
| --- | --- | --- |
| 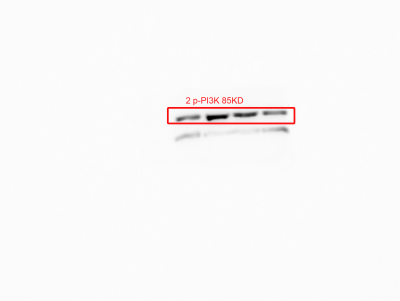 | 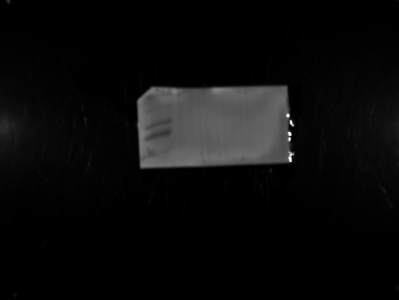 | 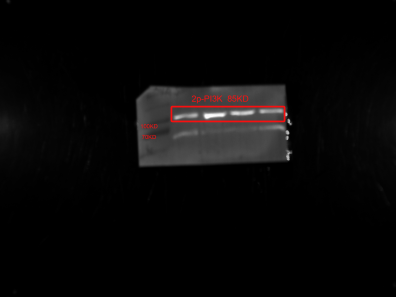 |
| 2gapdh |  |  |
| 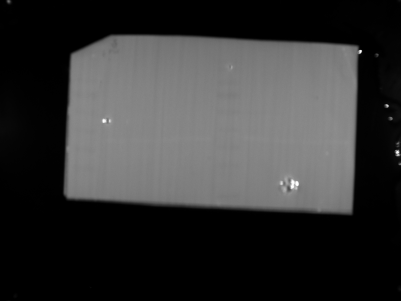 | 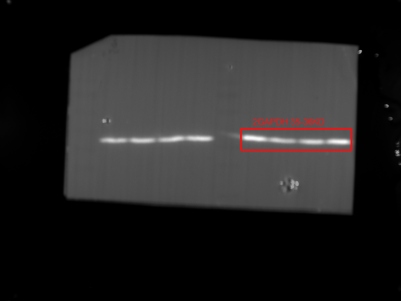 | 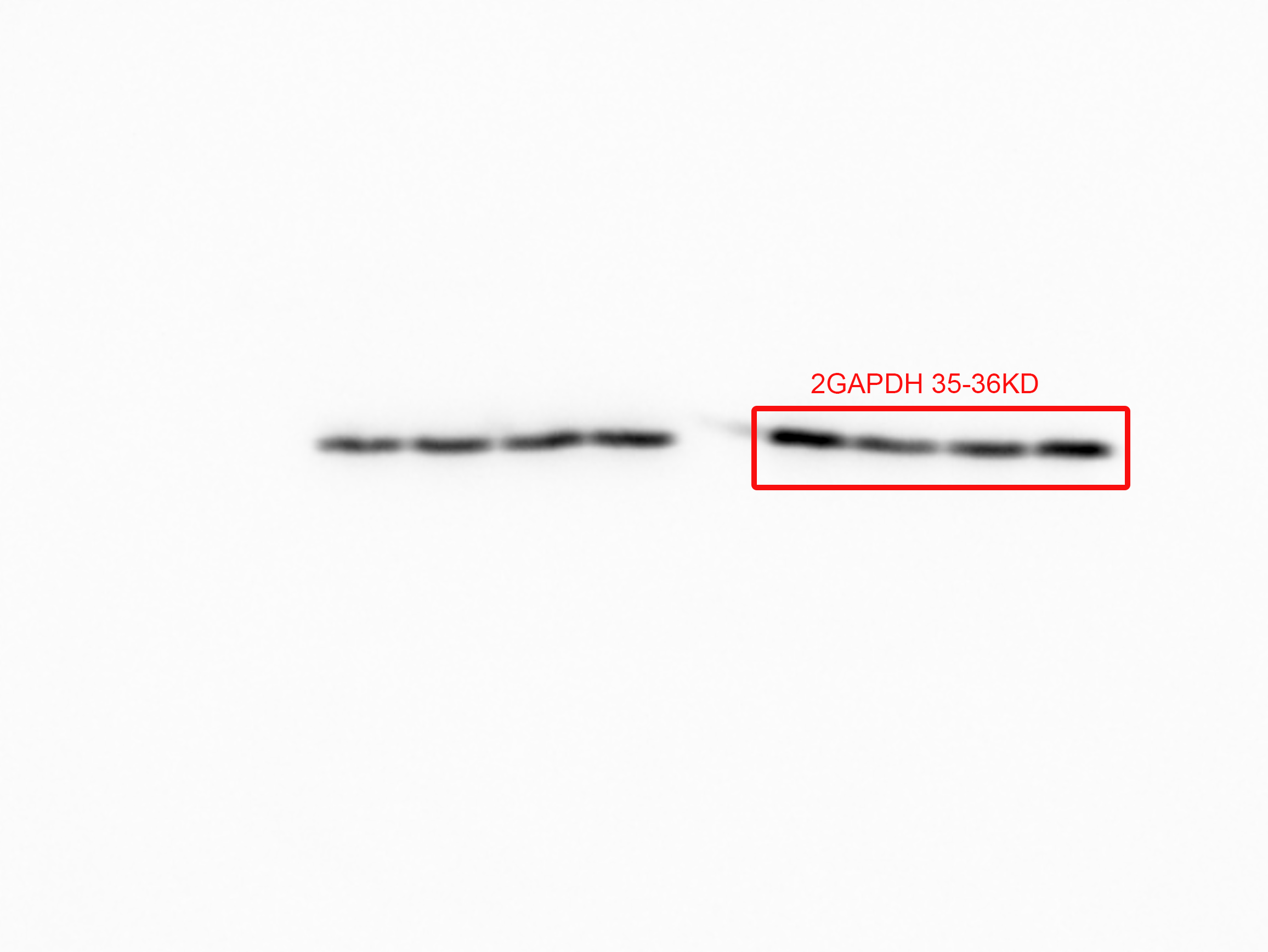 |

| 3p-PI3K |  |  |
| --- | --- | --- |
| 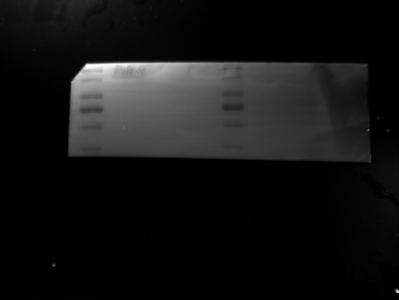 | 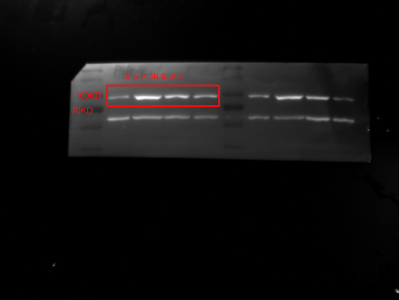 | 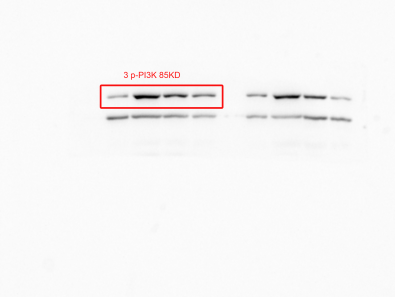 |
| 3gapdh |  |  |
| 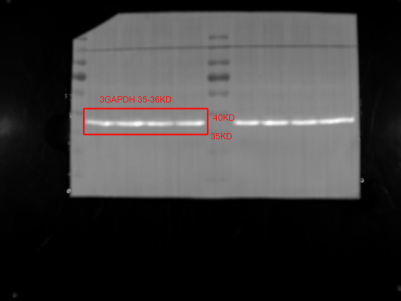 | 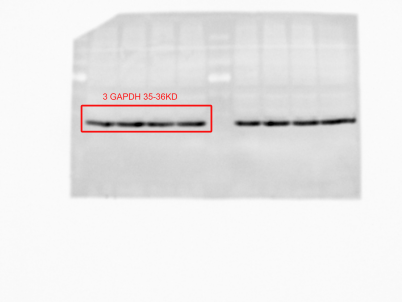 | 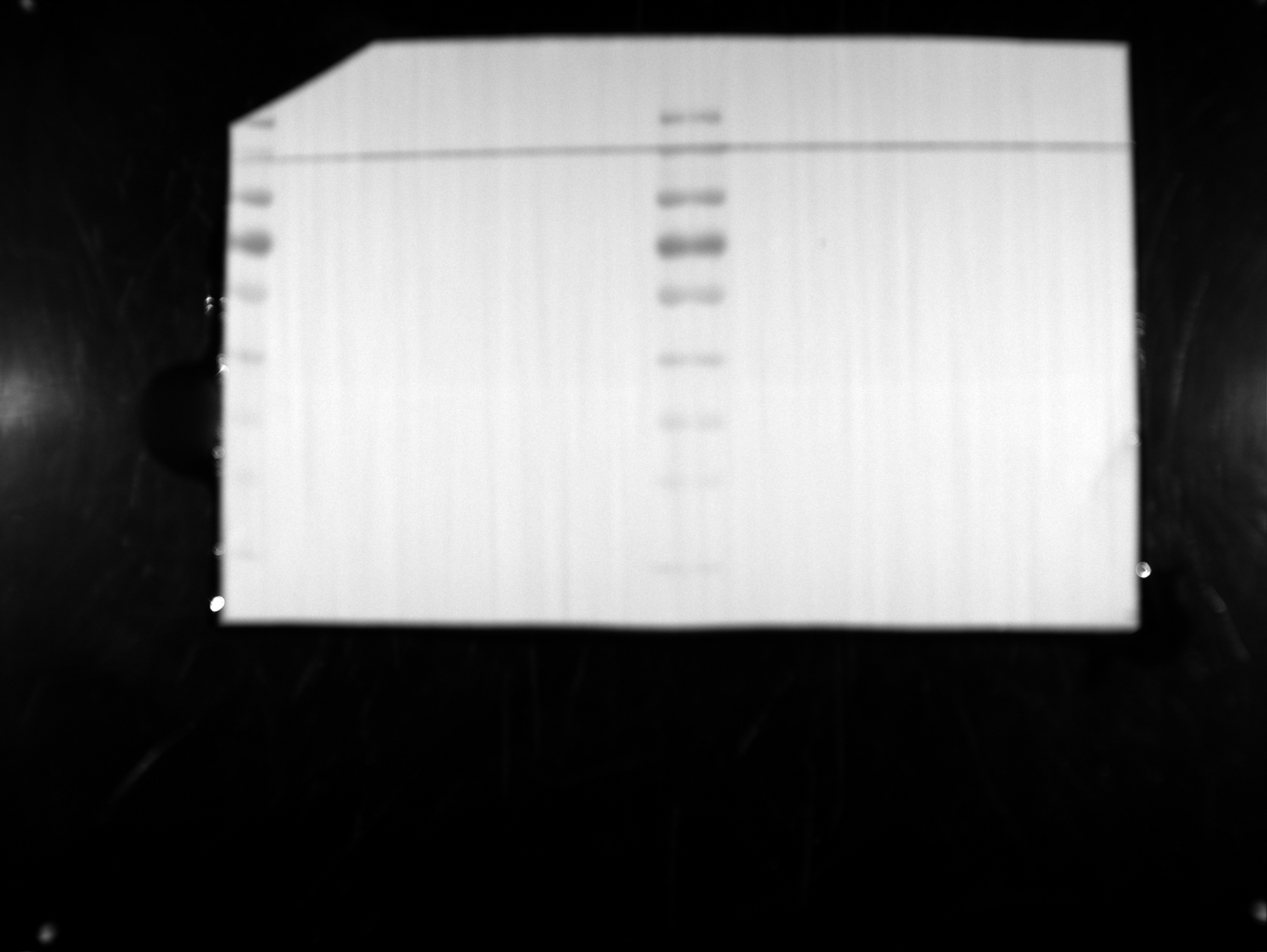 |

Supplement: Supplementary file 11 — Supplementary Information 11. [file 41598_2023_34060_MOESM11_ESM.docx]
