# Supplementary material for: Amifostine attenuates bleomycin-induced pulmonary fibrosis in mice through inhibition of the PI3K/Akt/mTOR signaling pathway
Source: Sci Rep. 2023 Jun 28;13:10485. doi: 10.1038/s41598-023-34060-8 (PMC10307827; doi:10.1038/s41598-023-34060-8)
Supplement: Supplementary file 12 — Supplementary Information 12. [file 41598_2023_34060_MOESM12_ESM.docx]

| 1PI3K |  | The image in the red frame on the left is the image shown by PI3K in Figure 6a |
| --- | --- | --- |
| 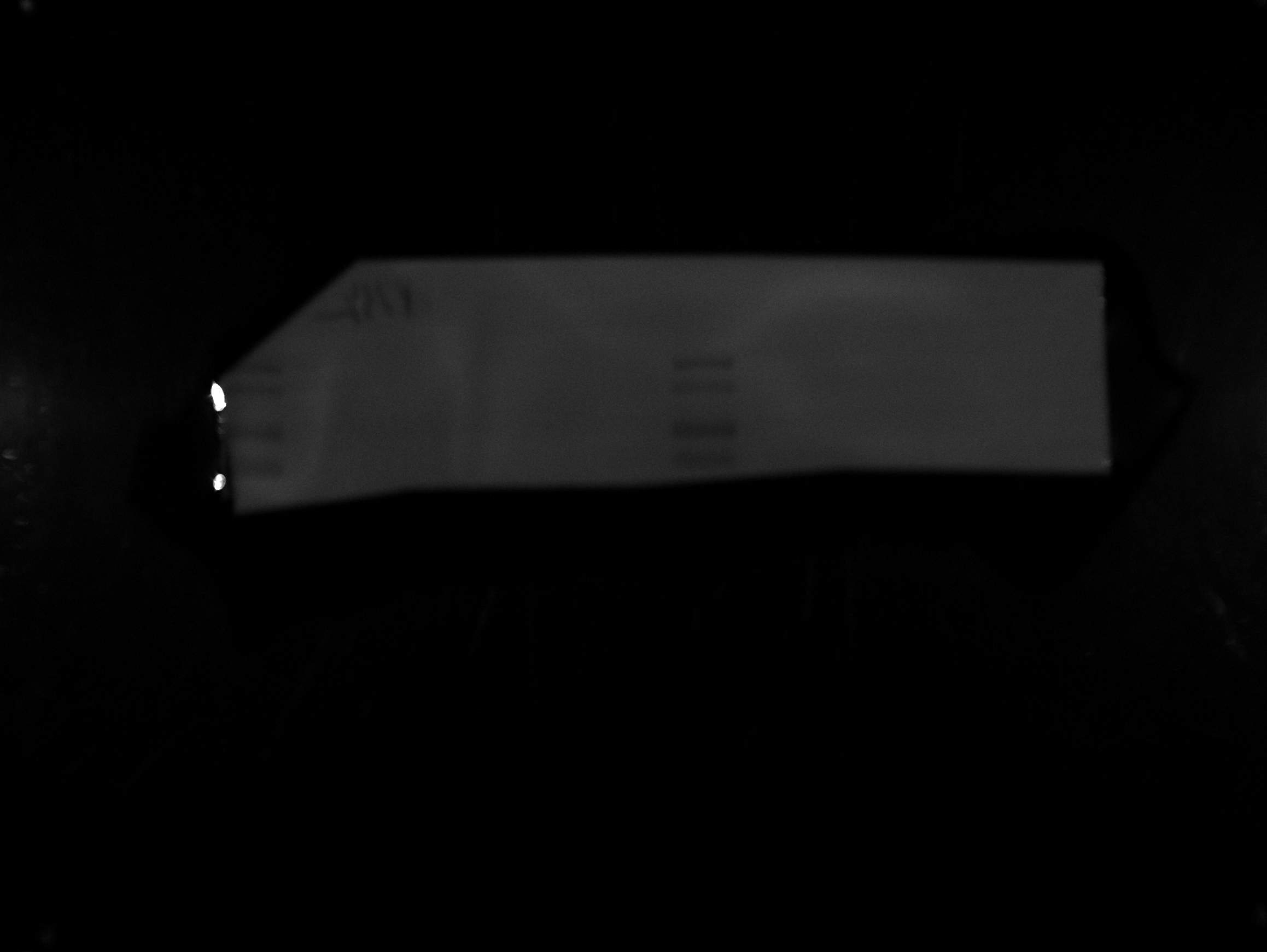 | 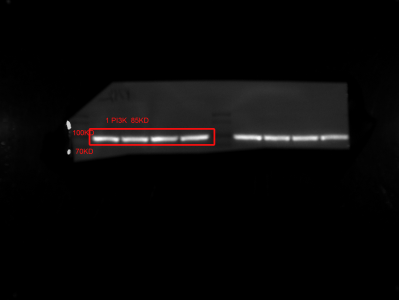 | 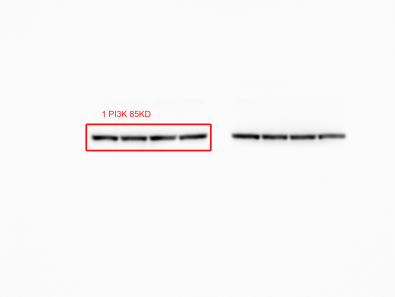 |
| 1gapdh |  |  |
| 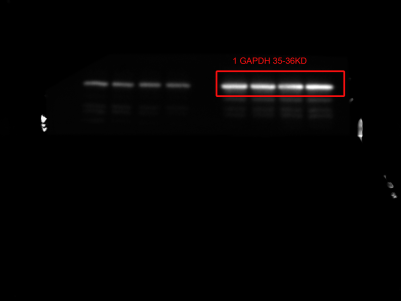 | 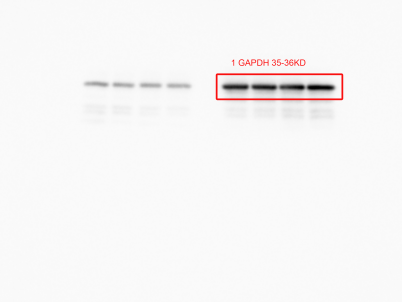 | 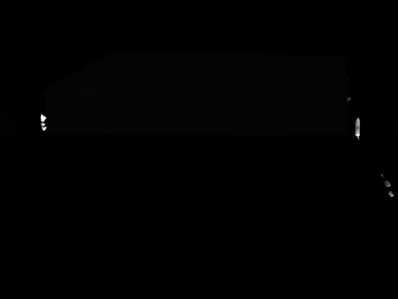 |

| 2PI3K |  |  |
| --- | --- | --- |
| 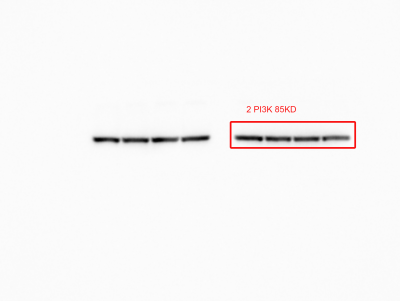 | 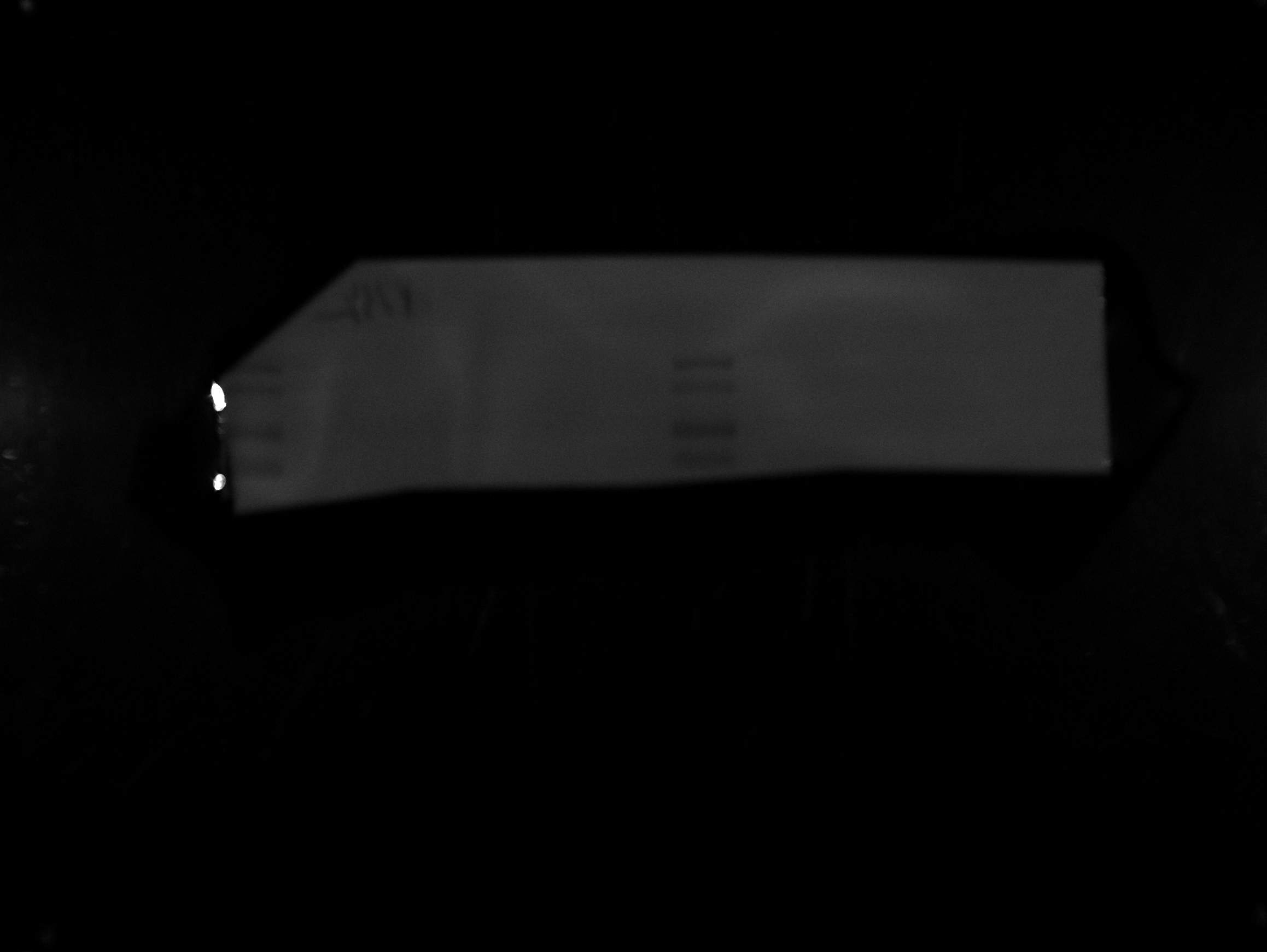 | 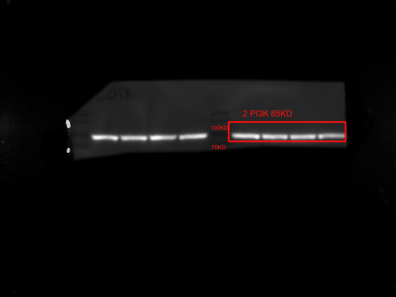 |
| 2gapdh |  |  |
| 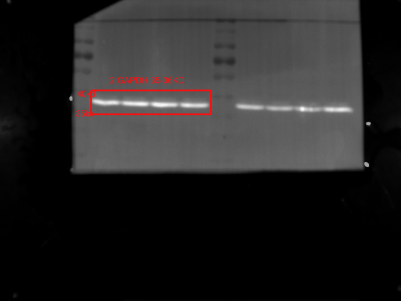 | 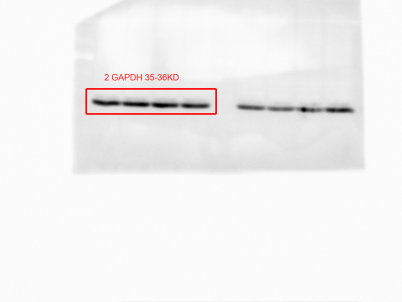 | 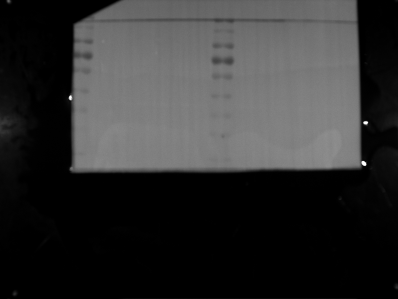 |

| 3PI3K | The strip on the right is the target band |  |
| --- | --- | --- |
| 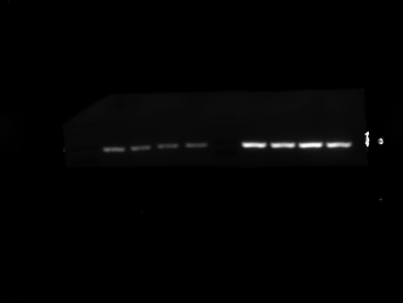 | 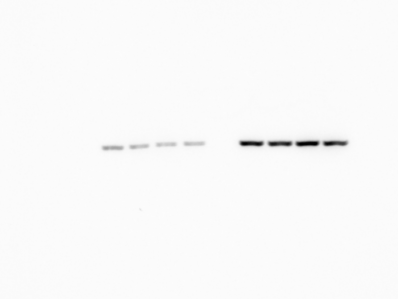 | 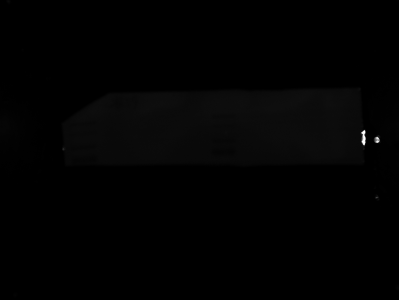 |
| 3gapdh |  |  |
| 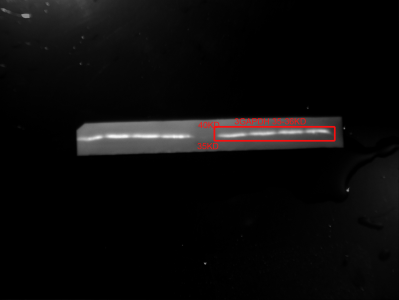 | 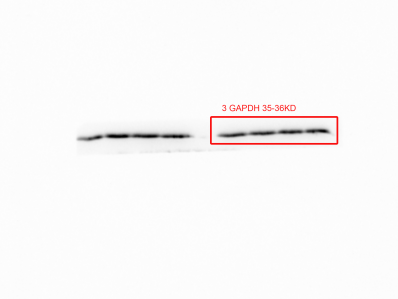 | 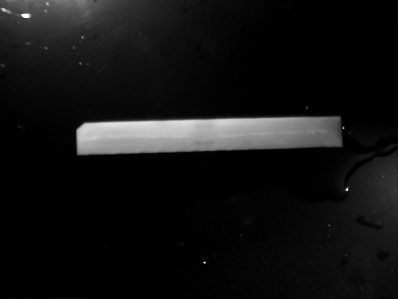 |
